# α-NiO/Ni(OH)_2_/AgNP/F-Graphene Composite for Energy Storage Application

**DOI:** 10.1021/acsomega.2c07322

**Published:** 2023-03-15

**Authors:** Su Young Ryu, Michael R. Hoffmann

**Affiliations:** Environmental Science & Engineering, Linde Laboratory, California Institute of Technology, Pasadena, California 91125, United States

## Abstract

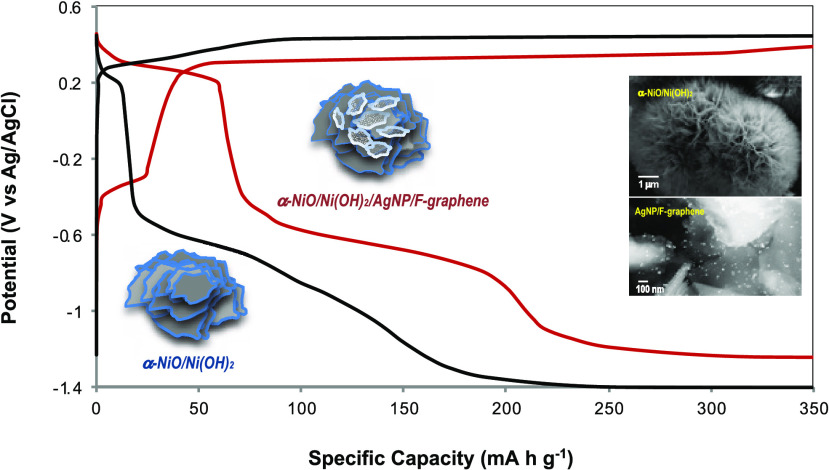

The α-NiO/Ni(OH)_2_/AgNP/F-graphene composite, which is silver nanoparticles preanchored on the surface of fluorinated graphene (AgNP/FG) and then added to α-NiO/Ni(OH)_2_, is investigated as a potential battery material. The addition of AgNP/FG endows the electrochemical redox reaction of α-NiO/Ni(OH)_2_ with a synergistic effect, resulting in enhanced Faradaic efficiency with the redox reactions of silver accompanied by the OER and the ORR. It resulted in enhanced specific capacitance (F g^–1^) and capacity (mA h g^–1^). The specific capacitance of α-NiO/Ni(OH)_2_ increased from 148 to 356 F g^–1^ with the addition of AgNP(20)/FG, while it increased to 226 F g^–1^ with the addition of AgNPs alone without F-graphene. The specific capacitance of α-NiO/Ni(OH)_2_/AgNP(20)/FG further increased up to 1153 F g^–1^ with a change in the voltage scan rate from 20 to 5 mV/s and the Nafion-free α-NiO/Ni(OH)_2_/AgNP(20)/FG composite. In a similar trend, the specific capacity of α-NiO/Ni(OH)_2_ increased from 266 to 545 mA h g^–1^ by the addition of AgNP(20)/FG. The performance of hybrid Zn–Ni/Ag/air electrochemical reactions by α-NiO/Ni(OH)_2_/AgNP(200)/FG and Zn-coupled electrodes indicates a potential for a secondary battery. It results in a specific capacity of 1200 mA h g^–1^ and a specific energy of 660 W h kg^–1^, which is divided into Zn–Ni reactions of ∼95 W h kg^–1^ and Zn–Ag/air reactions of ∼420 W h kg^–1^, while undergoing a Zn–air reaction of ∼145 W h kg^–1^.

## 1 Introduction

Energy storage devices have been developing rapidly due to a growing market for portable electronic devices including electric vehicles, mobile instruments, and large-scale energy storage applications. Typical energy storage devices depend on the conversion of chemical energy to electricity defined by electrochemical redox reactions in fuel cells, batteries, and electrochemical pseudocapacitors (i.e., supercapacitors). Supercapacitors (SCs) are useful components for portable electronic devices, providing high power densities, long life cycles, and short charging times. Despite relatively poor storage capacities, hybrid supercapacitors have attracted increasing attention over the last decade.^1−11^ The differences between SCs and batteries are due to their distinctly different energy storage mechanisms, although they have similar components such as electrodes, separators, and electrolytes. For example, Li-related batteries like Li-ion and Li-metal have high energy densities but low power densities. In contrast, SCs have high power densities with long-term cycle stability but low energy densities. A lot of research has focused on enhancing the specific energy of battery–supercapacitor hybrid (BSH) devices by modification of the electrode potential with optimizing material properties in a complementary strategy.^1−4^

The research field of supercapacitors has focused on replacing RuO_2_ with NiO, Co_3_O_4_, MnO_2_, Co(OH)_2_, or Ni(OH)_2_ as low-cost alternative materials for energy storage. Ni attracts our attention because it is abundant and environmentally friendly, and it has also proved its electrochemical properties through lots of research. For example, Ni(OH)_2_ undergoes electron transfer reactions, behaving like a capacitor that stores energy via redox (i.e., Faradaic) reactions. Ni(OH)_2_ has two polymorphs, α- and β-phases. β-phase consists of well-ordered stacked layers of Ni(OH)_2_, while α-Ni(OH)_2_ has randomly stacked and positively charged layers aligned along the c-axis. Exchangeable anions and water molecules are intercalated in the spaces between layers to provide overall electroneutrality in α-Ni(OH)_2_. Lee et al. reported the specific capacitance of α-Ni(OH)_2_, which is dependent on the size of the intercalated anions between layers (e.g., the largest anion of SO_4_^2–^ yields a low specific capacitance while giving the highest specific capacitance with the intercalation of the smallest anion of Cl^–^).^12^ It has been attributed to charge repulsion between the intercalated anion and OH^–^ in the alkaline electrolyte.^5^ In comparison, the electrochemical performance of the primary catalyst embodied in the electrode (EC) is dependent on the size and shape of the electrode materials. For example, α-Ni(OH)_2_ normally exhibits better electrochemical performance than β-Ni(OH)_2_. Batteries that are α-Ni(OH)_2_-based undergo a capacity decrease over charge–discharge cycles due to a phase transformation into the β-phase under alkaline conditions because of the greater thermodynamic stability of β-Ni(OH)_2_ compared to the α-phase Ni(OH)_2_.^13−15^ To retard the phase transformation from α to β phase, the metal ion dopants such as Co^3+^, Mn^3+^, Zn^2+^, Cd^2+^, and Al^3+^ are often added to stabilize the crystal structure of α-Ni(OH)_2_.^16−23^

On the other hand, the Faradaic property of the metal hydroxide is relatively low compared to the metal oxide due to an intrinsic poor electronic conductivity. The surface-modified α-NiO/Ni(OH)_2_ composite, which contains NiO partially, has been reported to have superior pseudocapacitive properties, indicating a relatively high Faradaic current with enhanced surface area compared to Ni(OH)_2_ itself.^15^

The fabrication of Ni(OH)_2_ electrodes normally requires an adhesive binder such as a conventional conducting polymer. However, it still gives low conductivity, resulting in inefficient electron transfer between the electroactive material and the current collector. Carbonaceous additives have been used to compensate for the low conductivities.^24,25^ Alternatively, inorganic colloidal nanocrystalline semiconductors have served as choice materials for electronic and optical devices due to their useful physical and electrochemical properties to improve conductivity. The nanocrystals could be readily mass-produced and used for device manufacturing, but still challenging to use for efficient electronic charge transfer from one to another in case the bulky organic surface ligand is capped on nanocrystals, which serves as an insulator. As reported, the electronic coupling between nanocrystals could be dramatically increased by the substitution of insulating organic molecules with novel inorganic molecules as reported previously.^26^

Metallic silver, Ag, can be used as an efficient bifunctional catalyst material in metal–air batteries since it often improves the performance of the OER and the ORR in aqueous alkaline media. The ORR occurs via direct or indirect processes. The direct ORR is a near-four-electron transfer reduction of O_2_ to OH^–^, while the indirect ORR is a two-electron transfer oxygen reduction, which is a lower efficiency pathway due to the disproportionation of HO_2_^–^.^27^ AgNPs have proven to be excellent electrocatalysts in the ORR as they catalyze the four-electron pathway, reducing oxygen to OH^–^.^28^ In addition, the halogenated graphene nanoplate like F-graphene has been reported to exhibit remarkable electrocatalytic activities for the oxygen reduction reaction (ORR) in alkaline electrolytes with long-term cycle stability. In detail, the halogenation into the sp^2^-hybridized orbitals of carbon regulates the surface electronic structure that plays a key role in ORR activities. As evidence, fluorine-functionalized graphene (F-graphene) has proven to be an even better four-electron ORR catalyst than Pt/C, which is known as a promising ORR catalyst.^33−36^

In this study, we developed the electrode α-NiO/Ni(OH)_2_/AgNP/FG, which is composed of α-NiO/Ni(OH)_2_ as the primary component with the silver nanoparticles anchored to fluorinated graphene (AgNP/F-graphene). This is likely a polymer binder-free electrode as-prepared successfully using a TiO_2_ nanogel instead of a conventional conductive polymer. The AgNPs anchored to F-graphene (AgNP/FG) promise an enhanced OER and ORR. This particular composite of α-NiO/Ni(OH)_2_/AgNPs/F-graphene results in an increased specific capacitance and specific capacity compared to the α-NiO/Ni(OH)_2_ core electrode.^29−36^

## 2 Experimental Section

### 2.1 Chemicals

Toray carbon paper treated with 120-PTFE was purchased from FuelcellsEtc. F-graphene was purchased from Cheap Tubes Inc. The commercial F-graphene has <30 wt % fluorine with a thickness of <2 nm and dimensions of 20 by 100 nm in a single layer and multiple sheets. Zn metal powder (<150 μm, 99.995% trace metals basis) was purchased from Sigma-Aldrich. A Zn foil of 0.1 mm thickness, 99.994% (metals basis), was purchased from Alfa Aesar, Puratronic. Zn foam, about 1 mm thickness, 99–99.9 % (metal basis), was purchased from American Elements.

### 2.2 Synthesis of α-NiO/Ni(OH)_2_

α-NiO/Ni(OH)_2_ was synthesized as follows: 1.46 g of Ni(NO_3_)_2_•6H_2_O and 0.25 g of sodium dodecyl sulfate (SDS) were dissolved in a solution of 25 mL of ethanol in 25 mL of Milli-Q water. Then, 3.0 g of urea was added and mixed via magnetic stirring until a homogeneous green transparent solution was obtained. The green transparent solution was transferred to a Teflon-lined autoclave and then heated at 110 °C for 15 h. The solid product was washed several times with each Milli-Q water and acetone and then dried in the oven at 80–100 °C overnight to obtain α-Ni(OH)_2_. The calcination of α-Ni(OH)_2_ was carried out at 260, 280, and 300 °C for 2 h.^15^ The mixed phase of α-NiO/Ni(OH)_2_ calcined at 280 °C was used as a major material for the current study of energy storage applications.

### 2.3 Synthesis of AgNP/F-Graphene and α-NiO/Ni(OH)_2_/AgNPs

AgNP/F-graphene was synthesized by 254 nm photoirradiation using a Hg lamp in 170 mL of isopropanol (IPA) solution dispersed with each of 20, 30, and 200 μM Ag(NO_3_) with 14 mg of F-graphene, respectively. The suspension was degassed by nitrogen (N_2_) purging for an hour to remove the dissolved oxygen before photoirradiation. The resulting products are denoted as AgNP(20)/FG, AgNP(30)/FG, and AgNP(200)/FG composites. The resulting particle sizes of AgNPs were dependent on the concentration of Ag(NO_3_) and the suspended amount of F-graphene.^74,75^

**Figure 1:**
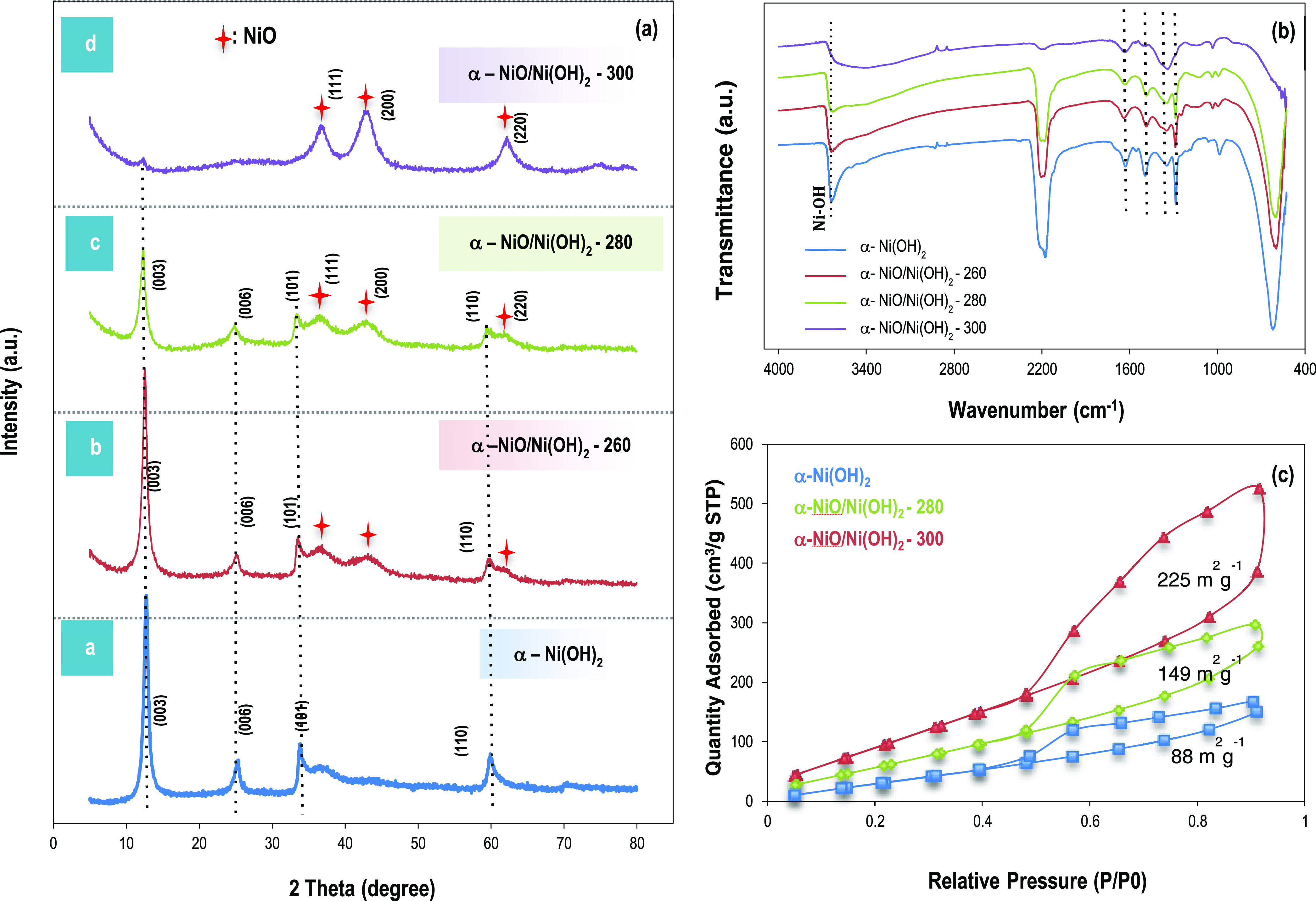
Characterization of α-Ni(OH)_2_ and α-NiO/Ni(OH)_2_ with XRD (a) and FTIR spectrum (b). Mixed phase of NiO and α-Ni(OH)_2_ was obtained by dehydration of α-Ni(OH)_2_ at *T* = 260, 280, and 300 ^o^C for 2 h and confirmed by the XRD pattern and FTIR spectra. (c) Nitrogen adsorption–desorption isotherm of α-NiO/Ni(OH)_2_-300 (=NiO), α-NiO/Ni(OH)_2_-280, and α-Ni(OH)_2_ (dried at <100 °C), indicating a hysteresis loop over the relative pressure range of 0.5–1.0, from which the BET surface area was determined to be 225, 149, and 88 m^2^g^–1^, respectively.

α-NiO/Ni(OH)_2_/AgNP composites were synthesized by photoirradiation (λ_254 nm_) of each 10 and 30 μM Ag(NO_3_) in 170 mL of IPA solutions dispersed with ∼3.3 mg of α-NiO/Ni(OH)_2_, denoted as α-NiO/Ni(OH)_2_/AgNP(10) and α-NiO/Ni(OH)_2_/AgNP(30), respectively. Dissolved O_2_ in the IPA solution was removed by nitrogen (N_2_) purging for 1 h before photoirradiation. The deposited AgNPs are shown in Figure 1b,c.

### 2.4 Synthesis of the TiO_2_ Nanogel

TiO_2_ was synthesized according to the following procedure. Titanium tetraisopropoxide, Ti(OCH(CH_3_)_2_)_4_, was diluted with isopropyl alcohol (IPA), and then it was added drop by drop into Milli-Q water in an M_H2O_/M_TTIP_ mass ratio of 110 and acidified with acetic acid to pH = 2. After complete hydrolysis, the suspension was heated at 90 °C for 4 h with vigorous stirring. TiO_2_ particles were collected by centrifugation that yields 5 wt % sol–gel conc., which is defined as a TiO_2_ nanoglue.^76,77^

### 2.5 Electrode Fabrication

The α-NiO/Ni(OH)_2_ electrode was prepared with the slurry of α-NiO/Ni(OH)_2_ and TiO_2_ nanogel mixture with an estimated mass ratio of ∼2.5:1 in ethanol and it was coated onto the carbon paper and then heat-treated at 250 °C for 1 h. The α-NiO/Ni(OH)_2_/AgNP electrode was prepared in a similar way as mentioned above. A α-NiO/Ni(OH)_2_/AgNP/F-graphene composite electrode was prepared using the same technique with a slurry mixture in an estimated mass ratio of 1:2.5:1–1.2 for AgNP/FG, α-NiO/Ni(OH)_2_, and TiO_2_ nanogel. Annealing was carried out at 250 °C for 1 hr. The proportion of AgNPs to the total mass of active material including α-NiO/Ni(OH)_2_, TiO_2_, and F-graphene is about 1 % for AgNP(20), ∼1.5% for AgNP(30), and ∼10 % for AgNP(200). The Zn/C electrode was prepared by applying a slurry coating on the carbon paper with a mixture of Zn powder, TiO_2_ nanogel, and F-graphene in a mass ratio of 3:5:0.15. After fabrication of α-NiO/Ni(OH)_2_/AgNP/FG electrodes, the surface obtained was a thin layer coated with as little Nafion as possible, followed by heat treatment at 130 °C for an hour. The schematic illustration of the fabrication and synthesis process of α-NiO/Ni(OH)_2_/AgNP/FG composite electrode is provided in Figure S1.

### 2.6 Analytical Methods

The crystal structure of α-NiO/Ni(OH)_2_ was confirmed with an X-ray diffractometer (XRD, PANalytical X-ray diffractometer, X’Pert Pro) equipped with Cu Kα radiation (λ = 1.50405 Å). The mesoporosity of α-NiO/Ni(OH)_2_ and α-Ni(OH)_2_ was confirmed by Brunauer–Emmett–Teller (BET) analysis, where the isotherms of nitrogen adsorption–desorption profile are observed with a distinct hysteresis loop in the high range of relative pressure between 0.5 and 1.0. The BET was carried out using a Micromeritics TriStar II surface area and porosity analyzer. The electrode surface morphology was investigated via SEM using a ZEISS 1550VP Field Emission Scanning Electron Microscope, with energy-dispersive X-ray spectroscopy (EDS, INGA Oxford) to identify the materials. Fourier transform infrared spectroscopy (FTIR) was carried out on a Nicolet iS50 spectrometer (Thermo Scientific, USA) with an integrated diamond crystal as an accessory. UV–vis diffuse reflectance spectra were determined using a Shimadzu UV-2101PC (dual-beam) equipped with an integrating sphere attachment (Shimadzu ISR-260), which is used to obtain reflection and transmittance measurements of liquid and solid samples. BioLogic Science potentiostats (VSP and SP 50) were used to acquire cyclic voltammograms and charge–discharge profiles.

## 3 Results and Discussion

### 3.1 Structural Characteristics of Active Materials and Composite Electrodes

α-NiO/Ni(OH)_2_ microspheres were prepared from Ni(NO_3_)_2_, sodium dodecyl sulfate (SDS), and urea. SDS was used as a structure-directing reagent, while urea was added to increase the pH, thus forcing precipitation of Ni(OH)_2_ in a water–ethanol mixture. In the presence of excess ammonia, a tetrahedral ammonia complex, Ni(NH_3_)_4_^2+^, was formed. The NH_3_ ligands of Ni(NH_3_)_4_^2+^ undergo hydrogen bonding with dodecyl sulfate, which eventually leads to a complete ligand exchange of ammonia by dodecyl sulfate anions. Under these conditions, Ni(OH)_2_ nanosheets were formed. With aging, the nanosheets incorporate dodecyl sulfate between the nanosheet layers, leading to the formation of microspheres due to Ostwald ripening.^15,37^

α-Ni(OH)_2_ was characterized by XRD, FTIR, and BET analysis. Dehydration of Ni(OH)_2_ was carried out with annealing at ≥260 °C, and it formed a mixed phase of NiO and α-Ni(OH)_2_ with a color change from green to dark gray (or dark green) depending on the temperature and time. The annealing at 300 °C for 2 h resulted in mostly NiO as identified with the XRD peaks at 38, 41, and 61, corresponding to the (111), (200), and (220) crystalline phases of NiO (Figure 1a–d). This is in accordance with the FTIR spectrum as no characteristic peak of O–H stretching was detected from >NiO–H at 3600 cm^–1^ (Figure 1b).^12^ The residual products were still detected at 1600, 1490, 1310, and 2170 cm^–1^, which are the bending or stretching modes of N–H, C–H, S=O, and SCN, respectively, and those are presumably the residue of decomposed dodecyl sulfate and urea.^38^ The TiO_2_ nanogel might catalyze the decomposition of SCN^–^ at the surface of α-NiO/Ni(OH)_2_ during the electrode fabrication process.

The surface area of α-Ni(OH)_2_ and α-NiO/Ni(OH)_2_ was confirmed by Brunauer–Emmett–Teller (BET) analysis. The isotherm of nitrogen adsorption–desorption has a hysteresis loop over the relative pressure range of 0.5 to 1.0 and resulted in a surface area of 225 m^2^g^–1^ for NiO and 149 m^2^g^–1^ for the mixed phase of α-NiO/Ni(OH)_2_. The surface area of α-Ni(OH)_2_ dried at <100 °C without a sintering process is obtained as 88 m^2^ g^–1^ (Figure 1c).

**Figure 2:**
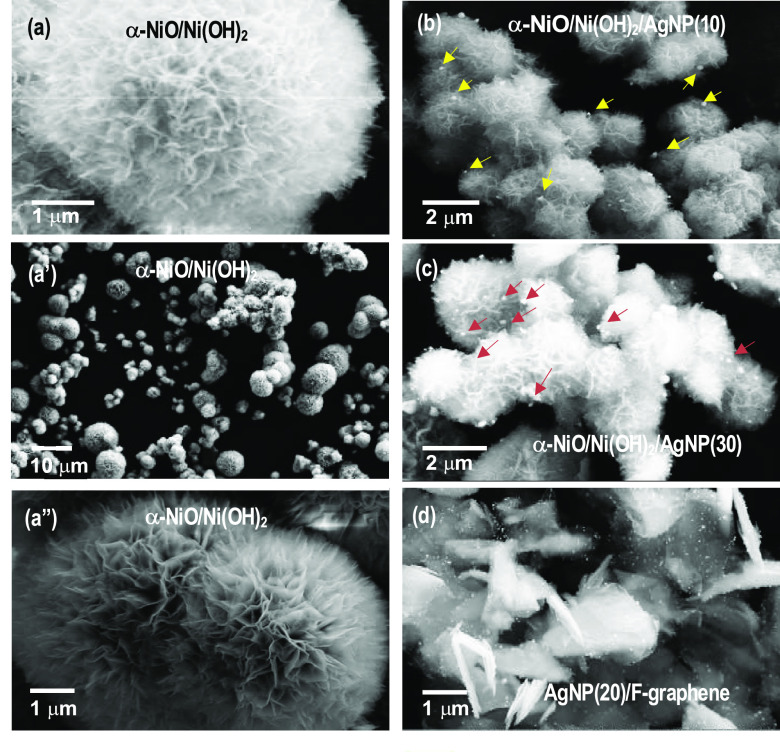
SEM images of the active materials. (a, a′, a″) α-NiO/Ni(OH)_2_ microspheres; (b) α-NiO/Ni(OH)_2_/AgNP(10): AgNP to α-NiO/Ni(OH)_2_ mole ratio of ∼1:2000; (c) α-NiO/Ni(OH)_2_/AgNP(30): AgNP to α-NiO/Ni(OH)_2_ mole ratio of ∼1:600 based on the concentration of AgNO_3_ and the amount of applied α-NiO/Ni(OH)_2_; (d) AgNP(20) anchored onto F-graphene.

SEM images of α-NiO/Ni(OH)_2_ and AgNPs are shown in Figure 2: (a, a′, a″) α-NiO/Ni(OH)_2_ microspheres, (b) α-NiO/Ni(OH)_2_/AgNP(10) composite, (c) α-NiO/Ni(OH)_2_/AgNP(30) composite, and (d) AgNP(20) anchored onto F-graphene. The synthesis of AgNPs on the surface of α-NiO/Ni(OH)_2_ was performed with 10 and 30 μmol of Ag(NO_3_) for each of α-NiO/Ni(OH)_2_/AgNP(10) and α-NiO/Ni(OH)_2_/AgNP(30) in isopropanol (IPA) solution, in which the α-NiO/Ni(OH)_2_ microspheres are dispersed. The particle size of AgNPs depends on the concentration of Ag(NO_3_) and the surface properties of the substrate. The resulting particle size ranges from a few tens to hundreds of nanometers, as shown in Figure 2b,c. On the other hand, AgNP(20) anchored to F-graphene has a particle diameter <20 nm (Figure 2d).

### 3.2 Evaluation of Electrochemical Properties

**Figure 3:**
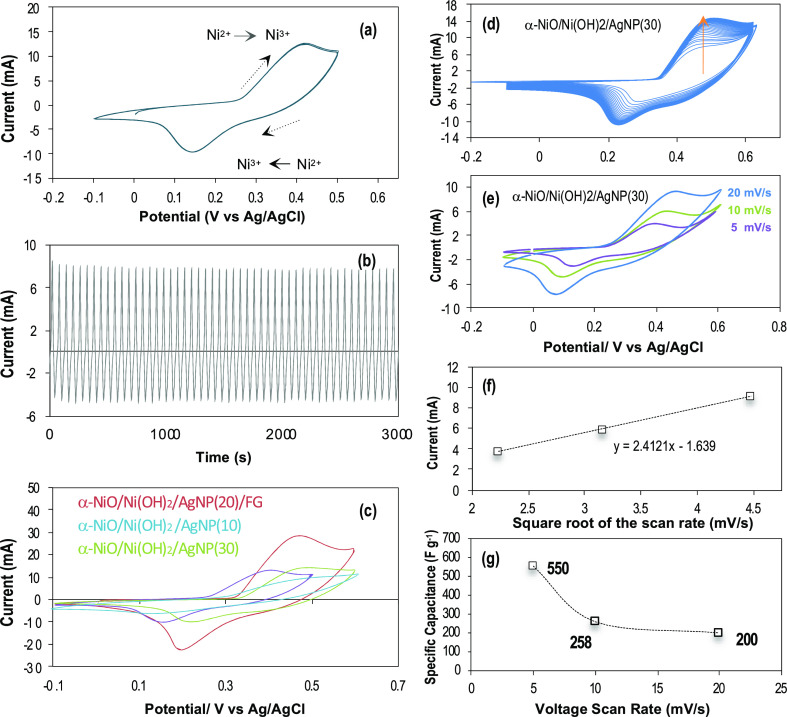
(a) CV of α-NiO/Ni(OH)_2_ defines a pseudocapacitive redox reaction. (b) CVs constantly preserved while repeating CV cycles for an hour. (c) Comparative CVs of α-NiO/Ni(OH)_2_, α-NiO/Ni(OH)_2_/AgNP(10), α-NiO/Ni(OH)_2_/AgNP(30), and α-NiO/Ni(OH)_2_/AgNP(20)/F-graphene obtained with a voltage scan rate of 20 mV/s in 1 M KOH. (d) CVs representing the activation process for the electrochemical redox reactions. (e) CVs corresponding to the voltage scan rates. (f) Anodic peak currents indicate to be linear to the square root of the voltage scan rates of 5, 10, and 20 mV/s. (g) Specific capacitance corresponding to the voltage scan rates. (f) and (g) resulted from (e).

α-NiO/Ni(OH)_2_ microspheres undergo several Faradaic redox reactions that eventually lead to the oxygen evolution reaction (i.e., the OER) as follows
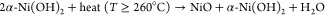
1
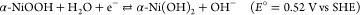
2
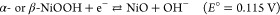
3
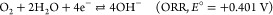
4
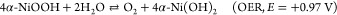
5Cyclic voltammetry (CV) curves for the electrodes were obtained under identical experimental conditions to determine the effect of AgNPs and F-graphene on the redox reactions of α-NiO/Ni(OH)_2_ (Figure 3). The electrodes were coated with a 5% Nafion solution to avoid a mass loss or migration of α-NiO/Ni(OH)_2_. The CV curve of α-NiO/Ni(OH)_2_ alone (Figure 3a) has a pair of redox peaks that is consistent with the oxidation of Ni^2+^ to Ni^3+^ at 0.41 V and the reduction of Ni^3+^ to Ni^2+^ at 0.13 V in a reversible process, which is reproducible while repeating CV cycles (Figure 3b). The redox potential of α-NiO/Ni(OH)_2_ is shifted to a higher potential region with increased current density upon the addition of AgNPs and AgNP/FG (Figure 3c). This is attributed to the electrochemical and physical properties of AgNPs and F-graphene. As a consequence, the specific capacitance increases in the following order: α-NiO/Ni(OH)_2_ (148 Fg^–1^) < α-NiO/Ni(OH)_2_/AgNP(30) (200 Fg^–1^) < α-NiO/Ni(OH)_2_/AgNP(10) (226 Fg^–1^) < α-NiO/Ni(OH)_2_/AgNP(20)/FG (356 Fg^–1^). The silver nanoparticles (AgNPs) in AgNP(20)/F-graphene are distributed quite evenly over the surface of fluorinated graphene with their particle size, *d* < 20 nm, as shown in SEM images. AgNPs anchored to F-graphene seem to maintain a nanosize characteristic owing to the two-dimensional hexagonal lattice structure of F-graphene having sp^2^-hybridized orbitals. In detail, the electrons located in a p_z_ orbital in graphene most likely form a π bond that hybridizes together to form a π- and π*- bands, allowing its exceptional electronic properties. In the case of fluorine-substituted graphene, some free-moving electrons in a p_z_ orbital interact with fluorine, leading to the formation of σ-bonds directed toward the z-axis, which is perpendicular to the graphene plane. Thus, the electronic and structural properties of the F-graphene may allow the silver nanoparticles to preserve quantized electrophysical properties.^39^ In contrast, the silver particles that formed on the surface of α-NiO/Ni(OH)_2_ are about 10 times larger than those on F-graphene, and they are oxidized and formed Ag_2_O clusters in strong alkali electrolytes. As a reason, the surface of α-NiO/Ni(OH)_2_ is partially hydrophobic due to the residual organic compounds incorporated into the product microspheres. In addition, the α-NiO/Ni(OH)_2_ microspheres have a relatively low BET surface area of ∼149 m^2^ g^–1^ compared to F-graphene with a surface area of >750 m^2^ g^–1^. The pretty high surface area of F-graphene most likely facilitates the formation and preservation of nanoparticulate silver (<20 nm) for AgNP(20)/F-graphene. The charge storage mechanism is reflected in the redox reactions of α-NiO/Ni(OH)_2_ in the KOH electrolyte; the reaction rate is dependent upon the concentration of KOH and the voltage scan rate of CV, as well as the distance between the anode and the cathode. For example, the CVs of α-NiO/Ni(OH)_2_/AgNP(30) indicate an activation process by OH^–^ diffusion through Nafion that eventually reached a steady-state (Figure 3d), and a function of the voltage scan rate shows a linear dependence of the peak currents on the square root of the voltage scan rate (Figure 3e,f). The specific capacitances corresponding to the voltage scan rates of 20, 10, and 5 mV s^–1^ are determined to be 200, 258, and 550 F g^–1^, respectively (Figure 3g). The specific capacitance that is inversely proportional to the voltage scan rate is due to a Faradaic redox reaction limited by the diffusion rate of OH^–^.

**Figure 4:**
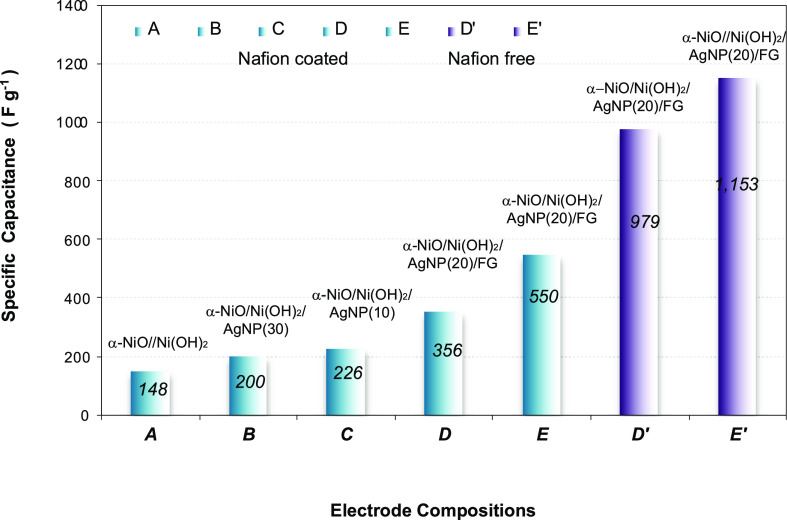
Representative specific capacitances of the electrodes. **A**, α-NiO/Ni(OH)_2_; **B**, α-NiO/Ni(OH)_2_/AgNP(30); **C**, α-NiO/Ni(OH)_2_/AgNP(10); **D** and **E**, α-NiO/Ni(OH)_2_/AgNP(20)/FG as a Nafion-coated electrode; **D′** and **E′**, a Nafion-free α-NiO/Ni(OH)_2_/AgNP(20)/FG electrode. **E** and **E′** were obtained at the voltage scan rate of 5 mV/s, while **A**, **B**, **C**, **D**, and **D′** were obtained at 20 mV/s.

A Nafion-free electrode was prepared using more TiO_2_ nanoglue to avoid any mass loss of the active material during electrolysis on the Nafion-free α-NiO/Ni(OH)_2_/AgNP(20)/FG electrode. Repeated CV cycles over 2 h confirm that the α-NiO/Ni(OH)_2_/AgNP(20)/FG electrode is stable without Nafion coating. The Nafion-free composite electrode exhibited improved current efficiency compared to the Nafion-coated electrode and resulted in an increased specific capacitance of up to 979 F g^–1^. In addition, a reduced voltage scan rate from 20 to 5 mV/s resulted in a further increased specific capacitance of up to 1153 F g^–1^. Figure 4 summarizes the specific capacitances of α-NiO/Ni(OH)_2_ family electrodes, which are dependent on the voltage scan rates with the composition of active materials, and the presence and absence of coated Nafion. Since Nafion affects the current efficiency, we minimized Nafion usage.

### 3.3 Specific Capacity

**Figure 5:**
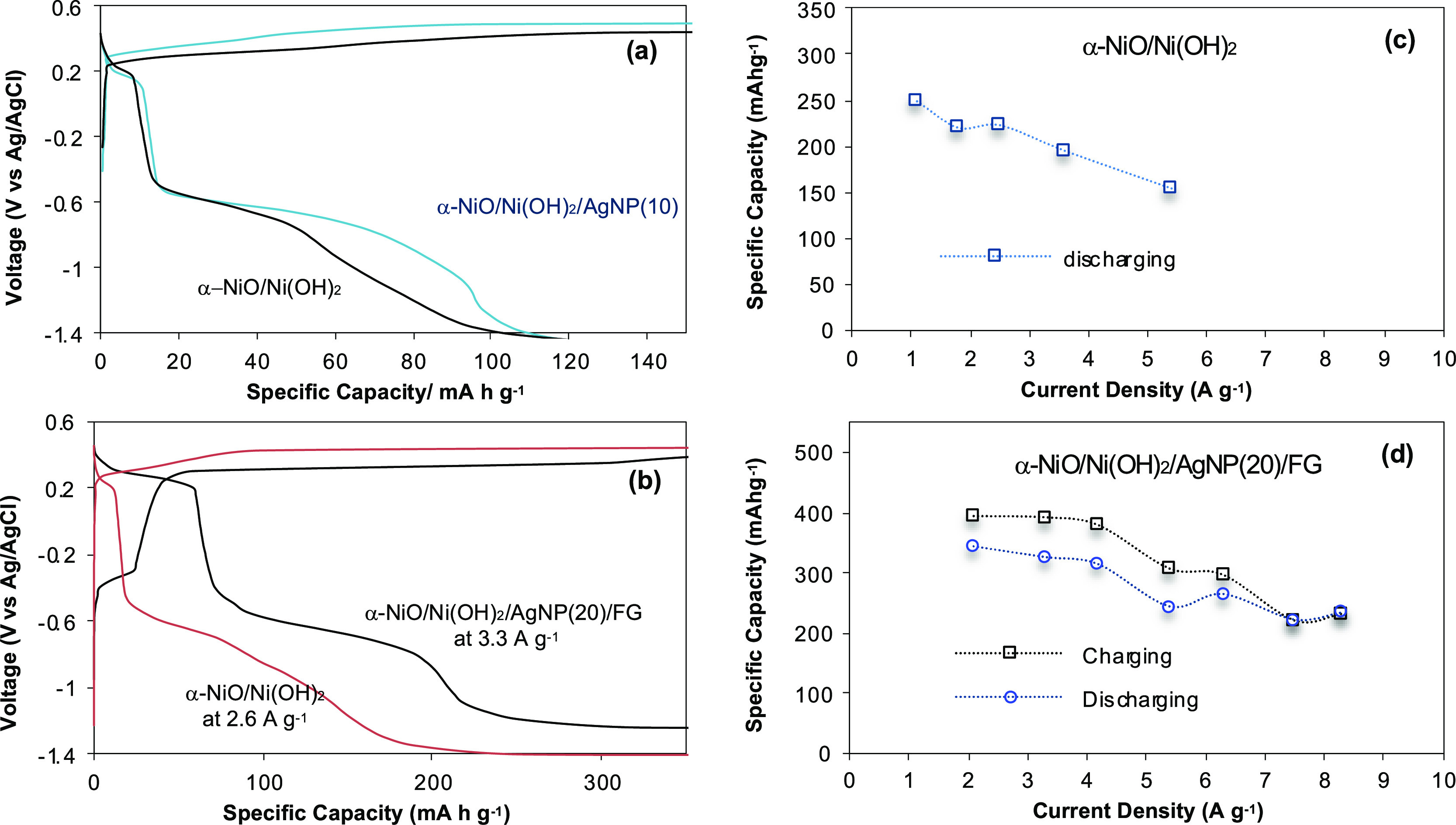
Comparative charge/discharge voltage profiles. (a) α-NiO/Ni(OH)_2_ and α-NiO/Ni(OH)_2_/AgNP(10); (b) α-NiO/Ni(OH)_2_ and α-NiO/Ni(OH)_2_**/**AgNP(20)/FG. The specific capacity of α-NiO/Ni(OH)_2_ (c) and α-NiO/Ni(OH)_2_/AgNP(20)/FG (d) corresponding to the current density. The mass of active materials for α-NiO/Ni(OH)_2_/AgNP(20)/FG is considered as α-NiO/Ni(OH)_2_ + AgNP(20).

The half-cell redox reaction of the α-NiO/Ni(OH)_2_/AgNP(20)/FG was explored based on the voltage profiles of charge/discharge cycles carried out using a standard three-electrode setup in 1.0 M KOH electrolyte. The discharge of α-NiO/Ni(OH)_2_ electrodes takes place initially at the potential of 0.29 V and −0.45 V vs Ag/AgCl, which is consistent with a sequential reduction of Ni^3+^ to Ni^2+^ and Ni^2+^ to Ni^0^. The electrode with added silver nanoparticles, AgNP(10), to the core α-NiO/Ni(OH)_2_ resulted in an extended plateau at −0.43 V (vs Ag/AgCl) (Figure 5a). In fact, it is possible for some of the nanosilver particles, Ag^0^, to be oxidized to Ag^+^ as the species, AgOH, at pH 14 (i.e., formation constants for AgOH, β_1_ = 10^2^ M^–1^ and Ag(OH)_2_^–^, β_2_ = 10^4^ M^–2^) in 1.0 M KOH. AgOH forms a dark brown silver(I) oxide (Ag_2_O, log *K*_sp_ = −7.7) via (2 AgOH ⇌ Ag_2_O + H_2_O, *K* = 10^5.75^). Thus, the extended plateau at −0.43 V (vs Ag/AgCl) is attributed to the reduction of Ag_2_O to Ag occurring at a potential close to the reduction of Ni^2+^ to Ni^0^. On the other hand, the α-NiO/Ni(OH)_2_/AgNP(20)/FG electrode exhibits extended plateaus at the potential of 0.29 and −0.45 V vs Ag/AgCl (Figure 5b). The AgNP(20)/FG, which has preanchored silver nanoparticles on the surface of F-graphene, seems to cause a synergistic effect on the overall electrochemical reactions due to the interaction of their electrophysical properties, not only between F-graphene and AgNP but also between AgNP/F-graphene and α-NiO/Ni(OH)_2_. The highest specific capacity with the α-NiO/Ni(OH)_2_ electrode is obtained at the current density of 1.1 A g^–1^, while the α-NiO/Ni(OH)_2_/AgNP(20)/FG reaches its highest specific capacity at the current density of 2.1 A g^–1^. It results in a 1.4-fold enhanced specific capacity compared to α-NiO/Ni(OH)_2_ itself (Figure 5c,d).

**Figure 6:**
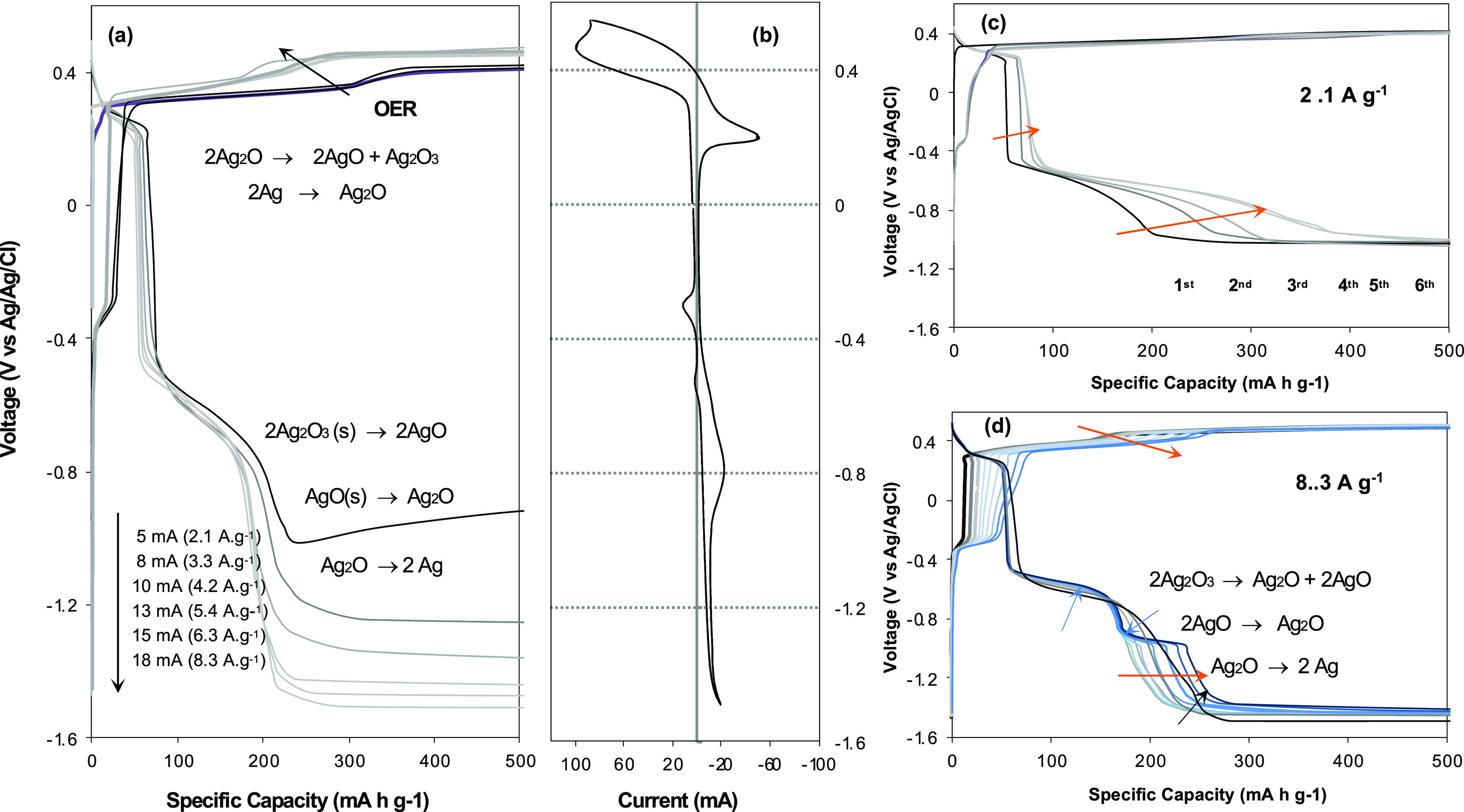
Charge/discharge voltage profiles and cyclic voltammetry obtained with α-NiO/Ni(OH)_2_/AgNP(20)/FG in 1 M KOH. (a) Charge/discharge voltage profiles at the current density of 2.1, 3.3, 4.2, 5.4, 6.3, and 8.3 Ag^–1^. (b) CV curve of α-NiO/Ni(OH)_2_/AgNP(20)/FG. (c) and (d) Charge/discharge voltage profiles developed by repeating charge/discharge cycles at the current density of 2.1 and 8.3 Ag^–1^, respectively. Each cycle consists of 30 min charge followed by 30 min discharge. The mass of active material is considered as AgNPs + α-NiO/Ni(OH)_2_.

The current density in the charge/discharge process is a crucial factor to achieve durable charge/discharge cycles, giving a reliable specific capacity. To explore how it affects electrochemical reactions, the charge/discharge voltage profiles of α-NiO/Ni(OH)_2_/AgNP(20)/FG are determined as a function of current density (Figure 6a). At the current density of 5.4 A g^–1^, the most obvious changes appear in the high voltage region where the OER takes place in the charging process and leads to facile Ag oxidation. The voltage profile is further explored via repeating cycles at the current densities of 2.1 and 8.3 A g^–1^, respectively (Figure 6c,d). The charge/discharge process at the relatively low current density of 2.1 A g^–1^ results in an increase of specific capacity from 345 to 545 mA h g^–1^ while repeating charge/discharge cycles. The discharge voltage profile reaches a plateau at −0.5 and −1.0 V (vs Ag/AgCl), where each reduction of AgO to Ag_2_O and Ag_2_O to Ag most likely takes place (Figure 6c). On the other hand, a repeating charge/discharge cycle at the relatively high current density of 8.3 A g^–1^ leads to a higher oxidation status of silver like Ag_2_O_3_ caused by the OER, which results in an additional reduction reaction of Ag_2_O_3_ to AgO along with AgO to Ag_2_O, and Ag_2_O to Ag at the subsequent discharging process (Figure 6d).^40−42^

A summary of the likely redox reactions involving α-NiO/Ni(OH)_2_ and AgNPs carried out at a low current density (e.g., 2.1 A g^–1^) in 1.0 M KOH is given below. The key AgNP reduction reactions include Ag^2+^ to Ag^+^ and Ag^+^ to Ag^0^ (eqs 6 and 7), while the nickel undergoes the redox reactions of eqs 9 and 10.
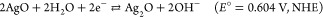
6
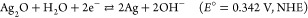
7
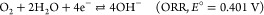
8
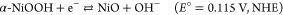
9
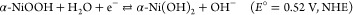
10

As mentioned above, the ORR in aqueous solutions proceeds by two alternative pathways: (i) a direct four-electron reduction, which occurs on a metal catalyst in general, or (ii) a two-electron reduction to peroxide, followed by the 2e^–^ reduction of H_2_O_2_/HO_2_^–^ to OH^–^ or alternative via a self-disproportionation reaction of 2HO_2_^–^ to yield 2 OH^–^ and O_2_, which is more common in alkali solution.^43^ AgNP is known to be an excellent electrocatalyst for the ORR that facilitates a direct four-electron reduction.^27,28,44−46^

At 8.3 A g^–1^, additional redox reactions take place upon charging and discharging along with possible electronic interactions between fluorinated graphene (FG) and the silver nanoparticles (eqs 11 and 13).^40^
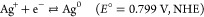
11
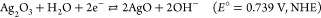
12
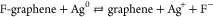
13

The addition of AgNP/F-graphene to α-NiO/Ni(OH)_2_ produced an increase in the specific capacity by a factor of 1.7. Moreover, the TiO_2_ nanoglue that is used to fabricate the composite electrode may have an impact on the specific capacity due to its dual functionality as a binder and high capacitive material.^47^

### 3.4 Application to Zn-Based Rechargeable Batteries

We investigated the charge/discharge voltage profiles of a hybrid Zn–Ni/Ag/air that is governed by two distinct pairs of Zn and α-NiO/Ni(OH)_2_/AgNP/FG.^48−52^ In 1.0 M KOH, the charge/discharge process of Zn–Ni/Ag/air involves the anodic redox reactions of Zn (eqs 14 and 15), complexation or hydrolysis (eqs 16 and 17), and dehydration or passivation (eq 18)
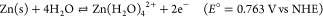
14
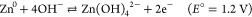
15
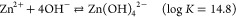
16
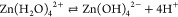
17
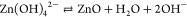
18When discharged, the reduction of silver and Ni occurs on the cathode, while Zn oxidation occurs on the anode. The overall electrolysis consists of reactions involving Zn–silver (eqs 19–21), Zn–Ni (eqs 22–24), and Zn–air (eqs 25–28) including Zn-ion battery reaction as follows
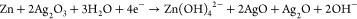
19
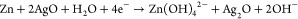
20
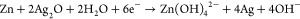
21
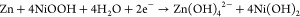
22
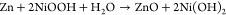
23
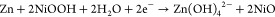
24anode:
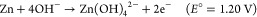
25fluid:
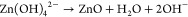
26cathode:
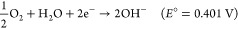
27overall:
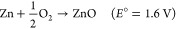
28In Zn–Ag/air batteries, the cathodic OER and ORR play pivotal roles, and the ORR is known to be a major limitation to performance efficiency due to its kinetics limitations.^53−57^ However, in this hybrid electrode system, it seems that the ORR overlapped with multiple silver reduction processes on AgNP/F-graphene.^43,45,58−61^ In addition, the localized electromagnetic field around AgNPs can induce a plasmonic resonance that accelerates the kinetics of the ORR.^62^ The ORR in the current study is clarified by the disappearance of O_2_ bubbles on the electrode surface during the discharge process. TiO_2_ nanoparticles and the carbon paper used as a current collector of the cathode may also contribute to the ORR, as extensively reported.^33,45,53−55,62−65^

On the other hand, the Zn-ion battery exhibits electrochemical reactions, which involve the reversible insertion/extraction of Zn^2+^ into/from the cathode as stripped out from the Zn anode in the discharging process, and the high overpotential for the hydrogen evolution reaction (HER) renders highly reversible zinc stripping and deposition in aqueous media.^66−72^ According to the principle of Zn-ion battery, the morphology of Zn could be considered as a major factor, determining Zn dissolution efficiency.

**Figure 7:**
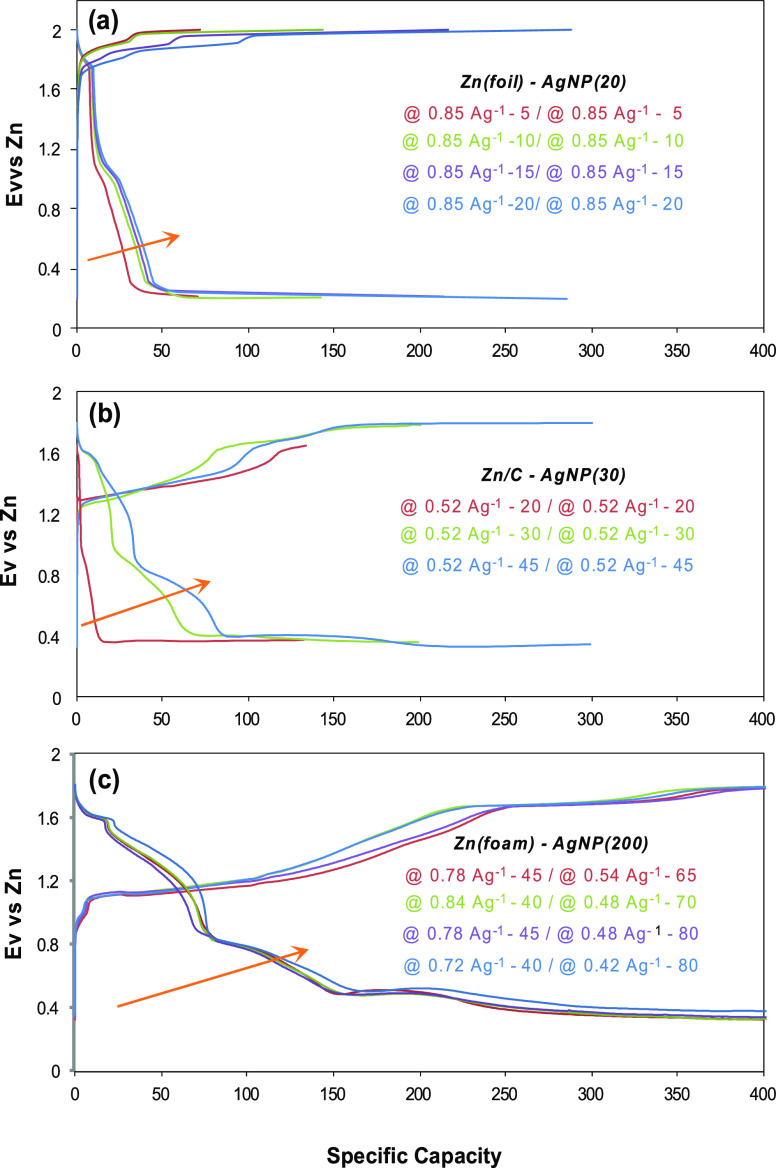
Charge/discharge voltage profiles of Zn-α-NiO/Ni(OH)_2_/AgNP/FG-coupled electrodes, which are dependent on the applied silver amount and Zn anode material. (a) Voltage profiles of Zn(foil)−α-NiO/Ni(OH)_2_/AgNP(20)/FG-coupled electrodes obtained at the current density of 0.85 Ag^–1^ with charging/discharging time variations of 5, 10, 15, and 20 min. (b) Voltage profiles of Zn/C-α-NiO/Ni(OH)_2_/AgNP(30)/FG-coupled electrodes at the current density of 0.52 Ag^–1^ with charging/discharging time variations of 20, 30, and 45 min. (c) Voltage profiles of Zn(foam)−α-NiO/Ni(OH)_2_/AgNP(200)/FG-coupled electrodes obtained with charging/discharging time and current density variation. The mass of active material is the total mass of AgNPs + F-graphene (FG) + α-NiO/Ni(OH)_2_ + TiO_2_. Zn mass is not included.

Figure 7 shows the charge/discharge voltage profiles dependent on silver amounts and Zn materials. The change in voltage profile appears over the potential range of 0.4–1.6 V, where the Zn–air and silver redox reactions take place. In the case of a Zn–air battery, the oxidation of Zn^0^ to Zn(OH)_4_^2–^ takes place close to 1.2 V, leading to the formation of ZnO. The reductions of Ag_2_O_3_, AgO, and Ag_2_O take place at *E* < 1.5 V (eqs 19–21). The discharge voltage is often inversely related to the current density.^73^ Discharge over the voltage range between 1.5 and 2 V involves primarily the Zn–Ni redox reactions including the reduction of α-NiOOH to α-Ni(OH)_2_ at *E*^o^ = 1.85 V and to α-NiO at *E*^o^ = 1.6 V. The voltage profile with Zn(foil)−α-NiO/Ni(OH)_2_/AgNP(20)/FG-coupled electrode exhibits two distinct voltage plateaus. The first one beginning at 1.8 V is attributed to the transformation of α-NiOOH to α-Ni(OH)_2_ and α-NiO, while the second plateau near 1.2 V is attributed to a reduction of Ag_2_O to Ag^0^ (AgNP) (Figure 7a). On the other hand, Figure 7b shows the charge/discharge voltage profiles with 1.5 times more Ag applied electrode (i.e., α-NiO/Ni(OH)_2_/AgNP(30)/FG) that is coupled with a Zn/C instead of a Zn(foil). Multivoltage plateaus indicate a charging/discharging time dependence. Similar changes are obtained with Zn(foam)−α-NiO/Ni(OH)_2_/AgNP(200)/FG-coupled electrodes (Figure 7c). In this case, AgNP(200) has approximately 10 times more silver than AgNP(20), and Zn(foam) has a larger surface area than Zn(foil). Since relatively low current density is applied to the discharging process, the discharge proceeds longer than the charging process in the manner of matching the charging/discharging specific capacity. Much expanded voltage plateaus are observed at the potentials of ∼1.5, 0.9, and ∼0.6 V, which are attributed to the reduction of Ag_2_O_3_ to AgO, AgO to Ag_2_O, and Ag_2_O to Ag^0^, respectively, accompanying the ORR. At the potential range where a Zn-ion battery reaction concomitantly initiates with Zn dissolution (stripping out) to Zn^2+^, the voltage plateaus must be influenced by a dissolution ability of Zn(foil), Zn/C, and Zn(foam).^66−72^ The CVs after charge/discharge cycles with Zn(foam)−α-NiO/Ni(OH)_2_/AgNP(200)/FG-coupled electrodes are shown in Figure S2.

The supplemental test upon a surplus charging effect is performed with a Zn(foil)−α-NiO/Ni(OH)_2_/AgNP(20)/FG-coupled electrodes. Two conditions are applied: one, charging/discharging at the identical condition for each cycle with increasing time in the sequence of 5, 10, 15, and 20 min (as shown in Figure S3a,b) and the other, unbalanced charging/discharging time process, which discharges for about 5 min in each cycle while increasing the charging time in the sequence of 5, 10, 15, and 20 min, as shown in Figure S3c,d, respectively. The unbalanced charging/discharging time process indicates a surplus charge that affects the next charge/discharge cycles, resulting in a more expanded voltage plateau compared to that at the identical condition.

The charge/discharge voltage profiles for the half-cell electrochemical reactions of α-NiO/Ni(OH)_2_/AgNP(200)/FG, which are applied to the lower current density for the discharge process, are shown in Figure S4. The voltage profile for the discharge process is defined as a portion of each reduction of Ag_2_O_3_ to AgO, AgO to Ag_2_O, and Ag_2_O to Ag, respectively, and each plateau expands corresponding to increased charge current density (see Figure S4a,c). Figure S4b is the half-cell electrochemical reaction of Zn(foam) as a reference. The CV (a) in Figure S4d is obtained after charge/discharge cycles for 123 h. It indicates a peak broadening in the voltage range between −1.2 and – 0.4 V Ag/AgCl, which is comparable to the CV (b) obtained prior to the charge/discharge cycles.

**Figure 8:**
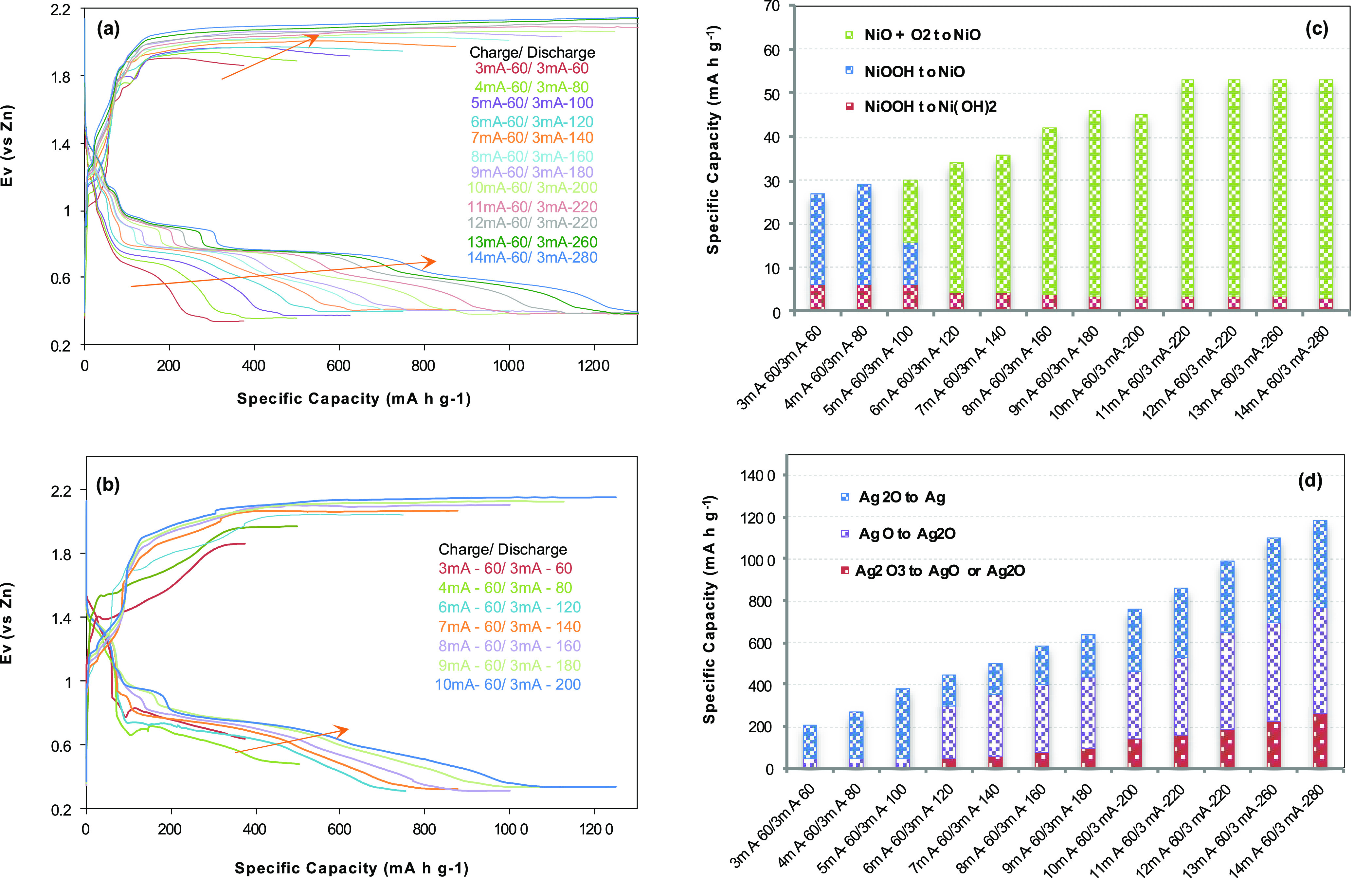
Charge/discharge voltage profiles and specific capacity for the Zn–Ni/Ag/air hybrid battery corresponding to charging current density. (a) Voltage profiles for 60 min charging with increasing current density from 0.375 A g^–1^ (3 mA) up to 1.75 Ag^–1^ (14 mA) in 0.125 A g^–1^ (1 mA) intervals, followed by discharging at 0.375 A g^–1^ (3 mA) with time variation from 60 to 280 min in the manner of matching the charge/discharge specific capacity. The charge/discharge cycle repeats three times at each current density and each third cycle is presented. (b) Charge/discharge voltage profiles obtained in the fresh electrolyte with the rinsed electrodes after the electrochemical reaction (a) for comparison. The representative specific capacity portions of each active material’s electrochemical performance in Zn–Ni/Ag/air hybrid batteries: (c) based on Zn–Ni-related electrochemical reactions, the estimated portion of Zn–Ni/air is about 53 mA h g^–1^; (d) based on Zn–Ag/air with Zn-ion electrochemical reactions adjusted as a sector of Ag_2_O_3_ to AgO or Ag_2_O, AgO to Ag_2_O, and Ag_2_O to Ag. The mass of active material is the total mass of AgNP + F-graphene + α-NiO/Ni(OH)_2_ + TiO_2_.

Figure 8shows the hybrid battery reactions of Zn–Ni/Ag/air including Zn ions. Figure 8a exhibits the charge/discharge voltage profiles for Zn (foam)−α-NiO/Ni(OH)_2_/AgNP(200)/FG-coupled electrodes, which charge for 60 min in each cycle with increasing current density from 0.375 Ag^–1^ (3 mA) up to 1.75 Ag^–1^ (14 mA) in 0.125 Ag^–1^ (1 mA) interval, followed by discharging at 0.375 Ag^–1^ (3 mA) in time variation from 60 to 280 min in the manner of matching the charge/discharge specific capacity. Figure 8b shows the voltage profiles obtained in a fresh 1 M KOH electrolyte with the rinsed electrode after the electrochemical reactions in Figure 8a for comparison. The representative specific capacities are presented in Figure 8c,d. As a result, the specific capacity based on the Zn–Ni electrochemical reactions is dependent on the charging current density, and the estimated specific capacity contribution of Zn–Ni is ∼ 53 mA h g^–1^. The specific capacity exhibited by the Zn–Ag/air battery reaction is distributed proportionally to the reduction of Ag_2_O_3_ to AgO, AgO to Ag_2_O, and Ag_2_O to Ag, which increases corresponding to the increased charging current density. The highest specific capacity is obtained as 1200 mA h g^–1^ at the charging current density of 1.75 Ag^–1^ (14 mA). Since Zn is not counted as active material due to the difficulty of measuring Zn mass involved in the battery reaction, it would result in a number of changes in the specific capacity and the specific energy presented herein.^62^

In this study, Zn–Ni/Ag/air hybrid battery reactions with the α-NiO/Ni(OH)_2_/AgNP/FG electrode are investigated as a potential secondary battery in which the role of AgNPs is to transform a primary Zn–air battery performance into a rechargeable battery with the aid of F-graphene and TiO_2_ nanoparticles.^50^ The specific energies for Zn-based batteries are reported in the following order: Zn–air (theoretical 1370 W h kg^–1^; operational 470 W h kg^–1^) > Zn–Ag (150–250 W h kg^–1^) > Zn–Ni (100 W h kg^–1^). However, the addition of silver nanoparticles in Zn–Ni and Zn–air batteries would be a trade-off between power density and energy density. A hybrid Zn–Ni/Ag/air battery reaction with the coupled electrodes of Zn (foam) and α-NiO/Ni(OH)_2_/AgNP(200)/FG yields a maximum specific energy of about 740 W h kg^–1^. This can be partitioned into contributions from Zn–Ni chemistry of ≤∼95 W h kg^–1^ and Zn–Ag/air chemistry of 500 W h kg^–1^. The Zn–air chemistry of 145 W h kg^–1^ is caused by slow redox reactions between Ag and F-graphene with the ORR on TiO_2_, while Zn oxidation occurs with unpleasant OER on the Zn electrode.^28^ This process is valuable to stabilize the electrodes with the completed redox reaction needed for the next charge/discharge cycle. It is worth noting that the interaction between AgNPs and fluorinated graphene seems to cause a self-discharge after Zn–Ag/air electrochemical reactions, and it is glaringly obvious corresponding to the increased current density, resulting in a decreased specific energy of up to 82 W h kg^–1^ as estimated.^33^ This is indicated in Figure S5A with a red arrow. As a result, the specific energy of 740 W h kg^–1^ contributed by each battery reaction with the Zn(foam)−α-NiO/Ni(OH)_2_/AgNP(200)/FG-coupled electrodes eventually results in ∼ 660 W h kg^–1^ that is applicable. The representative specific energy is presented in Figure S6 (refer to Table S1).

The overall discharge process can be broken down into a contribution from the Zn–Ni redox reactions that occur at the rates of 0.130 and 0.1025 (W h kg^–1^ s^–1^) for each redox reaction of Zn–Ni(OH)_2_ and Zn–NiO and a contribution from the Zn–Ag/air reactions at the distinguished rates of 0.085, 0.072, and 0.035 (W h kg^–1^ s^–1^) for Zn-Ag_2_O_3_, Zn-AgO, and Zn-Ag_2_O redox reactions, respectively Lastly, a contribution from Zn–air at a discharge rate of 0.04 W h kg^–1^ s^–1^ including a much slower discharge rate of <0.02 W h kg^–1^ s^–1^ from either of Ag-F redox or/and the reduction of F-graphene as estimated. The representative data are presented in Figure S5B.

## 4 Conclusions

In this study, we developed the electrode of α-NiO/Ni(OH)_2_/AgNP/FG that is composed of α-NiO/Ni(OH)_2_ as a primary component with the silver nanoparticles preanchored to fluorinated graphene (AgNP/FG). This is a polymer binder-free electrode as-prepared using a TiO_2_ nanogel instead of a conventional conductive polymer. The AgNPs anchored to F-graphene (AgNP/FG) promise an enhanced OER and ORR since the electrophysical properties of F-graphene play a pivotal role, allowing AgNPs to participate in multiredox reactions. The TiO_2_ nanogel used as glue improves the Coulombic efficiencies for the OER and the ORR as intrinsic electrocatalytic properties. This particular composite of α-NiO/Ni(OH)_2_/AgNP(20)/F-graphene results in enhanced specific capacitance (F g^–1^) and specific capacity (mA h g^–1^) compared to the α-NiO/Ni(OH)_2_ core electrode, resulting in increased specific capacitance from 148 to 356 F g^–1^ and the specific capacity from 266 to 545 mA h g^–1^. As for the specific capacitance, it is further increased up to 1153 F g^–1^ with a change of voltage scan rate from 20 to 5 mV/s and the Nafion-free α-NiO/Ni(OH)_2_/AgNP(20)/FG composite.

The performance of hybrid Zn–Ni/Ag/air electrochemical reactions by α-NiO/Ni(OH)_2_/AgNP(200)/FG and Zn-coupled electrode indicates a potential for a secondary battery. It results in a specific capacity of 1200 mA h g^–1^ and a specific energy of 660 W h kg^–1^, which is divided into Zn–Ni reactions of ∼95 W h kg^–1^ and Zn–Ag/air of ∼420 W h kg^–1^, while undergoing a Zn–air reaction of ∼145 W h kg^–1^.

## Supporting Information Available

Schematic illustration of the fabrication and synthesis process of active materials and electrodes (Figure S1); CVs and charge/discharge voltage profiles of the half-cell and the coupled electrodes of Zn(foam) and α-NiO/Ni(OH)_2_/AgNP(200)/FG with a specific capacity that is dependent on the current density (Figures S2–S4); and results of the specific energy (W h Kg^–1^), which are the plot of specific energy and the energy draining rates for the active species of Ni and Ag, and the portion of specific energy contributed by the electrochemical reaction of Zn–Ni, Zn–Ag/O_2_, Zn–air, as listed on Table S1 (Figures S5–S6) (PDF)

## References

[ref1] Wang Q.; Wen Z. H.; Li J. H. A Hybrid Supercapacitor Fabricated with a Carbon Nanotube Cathode and a TiO_2_-B Nanowire Anode. Adv. Funct. Mater. 2006, 16, 2141–2146. 10.1002/adfm.200500937.

[ref2] Wang Y.-G.; Xia Y.-Y. A New Concept Hybrid Electrochemical Supercapacitor: Carbon/LiMn_2_O_4_ Aqueous System. Electrochem. Commun. 2005, 7, 1138–1142. 10.1016/j.elecom.2005.08.017.

[ref3] Vlad A.; Singh N.; Rolland J.; Melinte S.; Ajayan P. M.; Gohy J. F. Hybrid Supercapacitor battery Materials for Fast Electrochemical Charge Storage. Sci. Rep. 2014, 4, 4315 10.1038/srep04315. 24603843 PMC3945924

[ref4] Miller J. R.; Simon P. Electrochemical Capacitors for Energy Management. Science 2008, 321, 651–652. 10.1126/science.1158736. 18669852

[ref5] Simon P.; Gogotsi Y.; Dunn B. Where do Batteries End and Supercapacitors Begin?. Science 2014, 343, 1210–1211. 10.1126/science.1249625. 24626920

[ref6] Simon P.; Gogotsi Y. Materials for Electrochemical Capacitors. Nat. Mater. 2008, 7, 845–854. 10.1038/nmat2297. 18956000

[ref7] Winter M.; Brodd R. What are Batteries, Fuel Cells, and Supercapacitors?. J. Chem. Rev. 2004, 104, 4245–4269. 10.1021/cr020730k. 15669155

[ref8] Mei H.; Mei Y.; Zhang S.; Xiao Z.; Xu B.; Zhang H.; Fan L.; Huang Z.; Kang W.; Sun D. Bimetallic-MOF Derived Accordion-like Ternary Composite for High-Performance Supercapacitors. Inorg. Chem. 2018, 57, 10953–10960. 10.1021/acs.inorgchem.8b01574. 30137967

[ref9] Bao Y.; Jia X.; Liu Lu.; Xiao Z.; Bu R.; Lv S.; Liu J.; Dang Z.; Zhang Q.; Wang L. Step-by-step Etching Strategy to Construct Multiple-shell Amorphous Co/Ni-(PO_4_)x(OH)y Hollow Polyhedron for Supercapacitor Application. J. Solid State Chem. 2021, 304, 122618 10.1016/j.jssc.2021.122618.

[ref10] Zhang J.; Deng Y.; Wu Y.; Xiao Z.; Liu X.; Li Z.; Bu R.; Zhang Q.; Sun W.; Wang L. Chemically Coupled 0D-3D Hetero-structure of Co_9_S_8_-Ni_3_S_4_ hollow Spheres for Zn-based Supercapacitors. Chem. Eng. J. 2022, 430, 132836 10.1016/j.cej.2021.132836.

[ref11] Jia X.; Wu Y.; Chi J.; Xiao Z.; Dang Z.; Zhang Q.; Li B.; Liu J.; Wang L. A VO_3_^-^-induced S^2-^- exchange strategy to controllably construct a sub-nano-sulfide-functionalized layered double hydroxide for an enhanced supercapacitor performance. Mater. Chem. Front 2022, 6, 2661–2669. 10.1039/D2QM00503D.

[ref12] Lee J. W.; Ko J. M.; Kim J.-D. Hierarchical Microspheres Based on α-Ni(OH)_2_ Nanosheets Intercalated with Different Anions: Synthesis, Anion Exchange, and Effect of Intercalated Anions on Electrochemical Capacitance. J. Phys. Chem. C 2011, 115, 19445–19454. 10.1021/jp206379h.

[ref13] Bastakoti B. P.; Huang H.-S.; Chen L.-C.; Kevin C.-W.; Yamauchi Y. Lock Copolymer Assisted Synthesis of Porous α-Ni(OH)_2_ Microflowers with High Surface Areas as Electrochemical Pseudo-capacitor Materials. Chem. Commun. 2012, 48, 9150–9152. 10.1039/c2cc32945j. 22854822

[ref14] Van der Ven A.; Morgan D.; Meng Y.; Ceder G. Phase Stability of Nickel Hydroxides and Oxyhydroxides. J. Electrochem. Soc. 2006, 153, A210–A215. 10.1149/1.2138572.

[ref15] Lee D. U.; Fu J.; Park M. G.; Liu H.; et al. Self-Assembled NiO/Ni(OH)_2_ Nanoflakes as Active Material for High-Power and High-Energy Hybrid Rechargeable Battery. Nano Lett. 2016, 16, 1794–1802. 10.1021/acs.nanolett.5b04788. 26854411

[ref16] Gong M.; Li Y.; Zhang H.; Zhang B.; Zhou W.; Feng J.; Wang H.; Liang Y.; Fan Z.; Liu J.; Dai H. Ultrafast High-Capacity NiZn Battery with NiAlCo-layered Double Hydroxide. Energy Environ. Sci. 2014, 7, 2025–2032. 10.1039/c4ee00317a.

[ref17] Provazi K.; Giz M. J.; Dall’Antonia L. H.; Crdoba de Torresi S. I. The Effect of Cd, Co, and Zn as Additives on Nickel Hydroxide Opto-Electrochemical Behavior. J. Power Sources 2001, 102, 224–232. 10.1016/S0378-7753(01)00819-9.

[ref18] Watanabe K.-i.; Koseki M.; Kumagai N. Effect of Cobalt Addition to Nickel Hydroxide as a Positive Material for Rechargeable Alkaline Batteries. J. Power Sources 1996, 58, 23–28. 10.1016/0378-7753(95)02272-4.

[ref19] Ramesh T. N.; Kamath P. V. The Effect of Cobalt on the Electrochemical Performance of β-nickel Hydroxide Electrodes. Electrochim. Acta 2008, 53, 8324–8331. 10.1016/j.electacta.2008.06.035.

[ref20] Li Y. W.; Yao J. H.; Liu C. J.; Zhao W. M.; Deng W. X.; Zhong S. K. Effect of Interlayer Anions on the Electrochemical performance of Al-substituted α-type nickel hydroxide electrodes. Int. J. Hydrogen Energy 2010, 35, 2539–2545. 10.1016/j.ijhydene.2010.01.015.

[ref21] Shaoan C.; Anbao Y.; Hong L.; Jianqing Z.; Chunan C. Effects of Barium and Cobalt on Electrochemical Performance of Nickel Hydroxide with Chemically Co-precipitated Zinc. J. Power Sources 1998, 76, 215–217. 10.1016/S0378-7753(98)00154-2.

[ref22] Wu M. Y.; Wang J. M.; Zhang J. Q.; Cao C. N. Effects of Coprecipitated Manganese on the Structure and Electrochemical Performance of Al-substituted α-Nickel Hydroxide. J. Solid State Electrochem. 2006, 10, 411–415. 10.1007/s10008-005-0023-9.

[ref23] Zhu W. H.; Ke J. J.; Yu H. M.; Zhang D. J. A Study of the Electrochemistry of Nickel hydroxide electrodes with various additives. J. Power Sources 1995, 56, 75–79. 10.1016/0378-7753(95)80011-5.

[ref24] Choi J.; Lee S.; Ha J.; Song T.; Paik U. Sol–gel Nanoglues for an Organic Binder-free TiO_2_ Nanofiber Anode for Lithium Ion Batteries. Nanoscale 2013, 5, 3230–3234. 10.1039/c3nr00157a. 23503470

[ref25] Yuan T.; Zhao B.; Cai R.; Zhou Y.; Shao Z. Electrospinning Based Fabrication and Performance Improvement of Film Electrodes for Lithium-ion Batteries Composed of TiO_2_ Hollow Fibers. J. Mater. Chem. 2011, 21, 15041–15048. 10.1039/c1jm11483b.

[ref26] Kovalenko M. V.; Scheele M.; Talapin D. V. Colloidal Nanocrystals with Molecular Metal Chalcogenide Surface Ligands. Science 2009, 324, 1417–1420. 10.1126/science.1170524. 19520953

[ref27] Goh F. W. T.; Liu Z.; Ge X.; Zong Y.; Du G.; Hor T. S. A. Ag nanoparticle-modified MnO_2_ nanorods catalyst for use as an air electrode in zinc-air battery. Electrochim. Acta 2013, 114, 598–604. 10.1016/j.electacta.2013.10.116.

[ref28] Kinoshita K., Carbon: Electrochemical and Physicochemical Properties; John Wiley & Sons: New York, 1988.

[ref29] Li B. J.; Cao H.; Shao J.; Zheng H.; Lu Y.; Yin J.; Qu M. Improved Performances of β-Ni(OH)_2_@Reduced-Graphene-Oxide in Ni-MH and Li-ion Batteries. Chem. Commun. 2011, 47, 3159–3161. 10.1039/c0cc04507a. 21279195

[ref30] Wang H.; Casalongue H. S.; Liang Y.; Dai H. Ni(OH)_2_ Nanoplates Grown on Graphene as Advanced Electrochemical Pseudocapacitor Materials. J. Am. Chem. Soc. 2010, 132, 7472–7477. 10.1021/ja102267j. 20443559

[ref31] Martins P. R.; Parussulo A. L. A.; Toma S. H.; Rocha M. A.; Toma H. E.; Araki K. Highly Stabilized Alpha-NiCo(OH)_2_ Nanomaterials for High Performance Device Application. J. Power Sources 2012, 218, 1–4. 10.1016/j.jpowsour.2012.06.065.

[ref32] Taibi M.; Ammar S.; Jouini N.; Fièvet F.; Molinie P.; Drillon M. Layered Nickel Hydroxide Salts: Synthesis, Characterization and Magnetic Behaviour in Relation to the Basal Spacing. J. Mater. Chem. 2002, 12, 3238–3244. 10.1039/B204087E.

[ref33] Guo J.; Zhang J.; Zhao H.; Fang Y.; Ming K.; Huang H.; Chen J.; Wang X. Fluorine-doped Graphene with an Outstanding Electrocatalytic Performance for Efficient Oxygen Reduction Reaction in Alkaline Solution. R. Soc. Open Sci. 2018, 5, 180925 10.1098/rsos.180925. 30473839 PMC6227960

[ref34] Zhang L. P.; Niu J. B.; Li M. T.; Xia Z. H. Catalytic Mechanisms of Sulfur-doped Graphene as Efficient Oxygen Reduction Reaction Catalysts for Fuel Cells. J. Phys. Chem. C 2014, 118, 3545–3553. 10.1021/jp410501u.

[ref35] Srinivasu K.; Swapan K. G. Transition Metal Decorated Graphyne: An Efficient Catalyst for Oxygen Reduction Reaction. J. Phys. Chem. C 2013, 117, 26021–26028. 10.1021/jp407007n.

[ref36] Gong K. P.; Du F.; Xia Z. H.; Durstock M.; Dai L. M. Nitrogen-doped Carbon Nanotube Arrays with High Electrocatalytic Activity for Oxygen Reduction. Science 2009, 323, 760–764. 10.1126/science.1168049. 19197058

[ref37] Kim J.-Y.; Kim K.-H.; Kim H.-K.; Park S.-H.; Roh K. C.; Kim K.-B. Template-Free Synthesis of Ruthenium Oxide Nanotubes for High-Performance Electrochemical Capacitors. ACS Appl. Mater. Interfaces 2015, 7, 16686–16693. 10.1021/acsami.5b04360. 26161814

[ref38] Wang H.; Tang Z.-Y.; Liu Y.-G.; Lee C.-S. Synthesis and behavior of Al-Stabilized α-Ni(OH)_2_. Trans. Nonferrous Met. Soc. China 2009, 19, 170–175. 10.1016/S1003-6326(08)60247-2.

[ref39] Cooper D. R.; D’Anjou B.; Ghattamaneni N.; Harack B.; Hilke M.; Horth A..; Majlis N.; Massicotte M.; Vandsburger L.; Whiteway E.; Yu V. Experimental Review of Graphene. ISRN Condens. Matter Phys. 2012, 2012, 1–56. 10.5402/2012/501686.

[ref40] Bailar J. C. The Oxidation States of Silver. J. Chem. Educ. 1944, 21, 523–525. 10.1021/ed021p523.

[ref41] Barradas R. G.; McDonnell D. B. Conditions for the Electrochemical Formation of Ag_2_O_3_ on Silver in Aqueous KOH solutions. Can. J. Chem. 1970, 48, 2453–2454. 10.1139/v70-415.

[ref42] Le S.; Zhang L.; Song X.; He S.; Yuan Z.; Liu F.; Zhang N.; Sun K.; Feng Y. Review-Status of Zinc-Silver Battery. J. Electrochem. Society 2019, 166, A2980–A2989. 10.1149/2.1001913jes.

[ref43] Neburchilov V.; Wang H.; Martin J. J.; Qu W. A Review on Air Cathodes for Zinc-air Fuel Cells. J. Power Sources 2010, 195, 1271–1291. 10.1016/j.jpowsour.2009.08.100.

[ref44] Spendelow J. S.; Wieckowski A. Electrocatalysis of oxygen reduction and small alcohol oxidation in alkaline media. Phys. Chem. Chem. Phys. 2007, 9, 2654 10.1039/b703315j. 17627310

[ref45] Guo J.; Hsu A.; Chu D.; Chen R. Improving oxygen reduction reaction activities on carbon-supported Ag nanoparticles in alkaline solutions. J. Phys. Chem. C 2010, 114, 4324 10.1021/jp910790u.

[ref46] Hacker V.; Wallnofer E.; Baumgartner W.; Schaffer T.; Besenhard J. O.; Schrottner H.; Schmied M. Carbon nanofiber-based active layers for fuel cell cathodes–preparation and characterization. Electrochem. Commun. 2005, 7, 377 10.1016/j.elecom.2005.02.009.

[ref47] Nam S. H.; Shim H.-S.; Kim Y.-S.; Dar M. A.; Kim J. G.; Kim W. B. Ag or Au Nanoparticle Embedded One-Dimensional Composite TiO_2_ Nanofibers Prepared via Electrospinning for Use in Lithium-Ion Batteries. ACS Appl. Mater. Interfaces 2010, 2, 2046–2052. 10.1021/am100319u.

[ref48] Latimer W. M. The Oxidation States of the Elements and Their Potentials in Aqueous Solutions, 2nd ed.; Prentice-Hall, Inc: Englewood Cliffs, NJ, 1952.

[ref49] Mizazzo G.; Caroli S. Tables of Standard Electrode Potentials; Wiley-Interscience: New York, 1978.

[ref50] Bard A. J.; Parsons R.; Jordan J. Standard Potentials in Aqueous Solution; Marcel Dekker, Inc: New York, 1985.

[ref51] Parker J. F.; Chervin C. N.; Pala I. R.; Machler M.; Burz M. F.; Long J. W.; Robinson D. R. Rechargeable Nickel-3D Zinc Batteries: An Energy-dense, Safer Alternative to Lithium-ion. Science 2017, 356, 415–418. 10.1126/science.aak9991. 28450638

[ref52] Yin L.; Scharf J.; Ma J.; Doux J.-M.; Redquest C.; Le V. L.; Yin Y.; Ortega J.; Wei X.; Wang J.; Meng Y. S. High Performance Printed AgO-Zn Rechargeable Battery for Flexible Electronics. Joule 2021, 5, 228–248. 10.1016/j.joule.2020.11.008.

[ref53] Steele B. C. H.; Heinzel A. Materials for Fuel-Cell Technologies. Nature 2001, 414, 345–352. 10.1038/35104620. 11713541

[ref54] Cheon J. Y.; Kim J. H.; Kim J. H.; Goddeti K. C.; Park J. Y.; Joo S. H. Intrinsic Relationship Between Enhanced Oxygen Reduction Reaction Activity and Nanoscale Work Function of Doped Carbons. J. Am. Chem. Soc. 2014, 136, 8875–8878. 10.1021/ja503557x. 24911055

[ref55] Zhang P.; Sun F.; Xiang Z. H.; Shen Z. G.; Yun J.; Cao D. P. ZIF-Derived in situ Nitrogen-Doped Porous Carbons as Efficient Metal-Free Electrocatalysts for Oxygen Reduction Reaction. Energy Environ. Sci. 2014, 7, 442–450. 10.1039/C3EE42799D.

[ref56] Li Y.; Dai H. Recent Advances in Zinc–Air Batteries. Chem. Soc. Rev. 2014, 43, 5257–5275. 10.1039/C4CS00015C. 24926965

[ref57] Chang C. C.; Lee Y. C.; Liao H. J.; Kao Y. T.; An J. Y.; Wang D. Y. Flexible Hybrid Zn–Ag/Air Battery with Long Cycle Life. ACS Sustainable Chem. Eng. 2019, 7, 2860–2866. 10.1021/acssuschemeng.8b06328.

[ref58] Chen Z.; Higgins D.; Yu A.; Zhang L.; Zhang J. A Review on Non-Precious Metal Electrocatalysts for PEM Fuel cells. Energy Environ. Sci. 2011, 4, 3167–3192. 10.1039/c0ee00558d.

[ref59] Tian J. Q.; Ning R.; Liu Q.; Asiri A. M.; Al-Youbi A. O.; Sun X. Three-Dimensional Porous Supramolecular Architecture from Ultrathin g-C3N4 Nanosheets and Reduced Graphene Oxide: Solution Self-Assembly Construction and Application as a Highly Efficient Metal-Free Electrocatalyst for Oxygen Reduction Reaction. ACS Appl. Mater. Interfaces 2014, 6, 1011–1017. 10.1021/am404536w. 24377299

[ref60] Xiao M. L.; Zhu J. B.; Feng L. G.; Liu C. P.; Xing W. Meso/Macroporous Nitrogen-Doped Carbon Architectures with Iron Carbide Encapsulated in Graphitic Layers as an Efficient and Robust Catalyst for the Oxygen Reduction Reaction in Both Acidic and Alkaline Solutions. Adv. Mater. 2015, 27, 2521–2527. 10.1002/adma.201500262. 25757871

[ref61] Zhang S.; Oms O.; Hao L.; Liu R.; Wang M.; Zhang Y.; He H.-Y.; Dolbecq A.; Marrot J.; Keita B.; Zhi L.; Mialane P.; Li B.; Zhang G. High Oxygen Reduction Reaction Performances of Cathode Materials Combining Polyoxometalates, Coordination Complexes, and Carbonaceous Supports. ACS Appl. Mater. Interfaces 2017, 9, 38486–38498. 10.1021/acsami.7b10989. 29035505

[ref62] Liu S.; Qin X. Preparation of a Ag–MnO_2_/Graphene Composite for the Oxygen Reduction Reaction in Alkaline Solution. RSC Adv. 2015, 5, 15627–15633. 10.1039/C5RA00280J.

[ref63] How G. T. S.; Pandikumar A.; Ming H. N.; Ngee L. H. Highly Exposed {001} Facets of Titanium Dioxide Modified with Reduced Graphene Oxide for Dopamine Sensing. Sci. Rep. 2014, 4, 5044 10.1038/srep05044. 24853929 PMC4031468

[ref64] Danilov F. I.; Tsurkan A. V.; Vasileva E. V.; Protsenko V. Electrocatalytic Activity of Composite Fe/TiO_2_ Electrodeposits for Hydrogen Evolution Reaction in Alkaline Solutions. Int. J. Hydrogen Energy 2016, 41, 7363–7372. 10.1016/j.ijhydene.2016.02.112.

[ref65] Bonanni A.; Ambrosi A.; Chua C. K.; Pumera M. Oxidation Debris in Graphene Oxide is Responsible for its Inherent Electroactivity. ACS Nano 2014, 8, 4197–4204. 10.1021/nn404255q. 24783949

[ref66] Zhang X. G., Corrosion and Electrochemistry of Zinc; Springer: US, 1996.

[ref67] Xu C.; Li B.; Du H.; Kang F. Energetic Zinc Ion Chemistry: The Rechargeable Zinc Ion Battery. Angew. Chem., Int. Ed. 2012, 51, 933–935. 10.1002/anie.201106307. 22170816

[ref68] Zhang Q.; Luan J.; Tang Y.; Ji X.; Wang H. Interfacial Design of Dendrite-free Zinc Anodes for Aqueous Zinc-Ion Batteries. Angew. Chem., Int. Ed. 2020, 59, 13180–13191. 10.1002/anie.202000162. 32124537

[ref69] Kundu D.; Vajargah S. H.; Wan L.; Adams B.; Gast D. P.; Nazar L. F. Aqueous Vs. Nonaqueous Zn-ion Batteries: Consequences of the Desolvation Penalty at the Interface. Energy Environ. Sci. 2018, 11, 881 10.1039/C8EE00378E.

[ref70] Zhou M.; Chen Y.; Fang G.; Liang S. Electrolyte/electrode Interfacial Electrochemical Behaviors and Optimization Strategies in Aqueous Zinc-ion Batteries. Energy Storage Mater. 2022, 45, 618–646. 10.1016/j.ensm.2021.12.011.

[ref71] Sambandam B.; Soundharrajan V.; Kim S.; Alfarugi M. H.; Jo J.; Kim S.; Mathew V.; Sun Y.; Kim J. Aqueous Rechargeable Zn-ion Batteries: an Imperishable and High-energy Zn_2_V_2_O_7_ Nanowire Cathode through Intercalation Regulation. J. Mater. Chem. A 2018, 6, 3850–3856. 10.1039/C7TA11237H.

[ref72] Shin J.; Lee J.; Park Y.; Choi J. W. Aqueous Zinc Ion Batteries: Focus on Zinc Metal Anodes. Chem. Sci. 2020, 11, 2028–2044. 10.1039/D0SC00022A. 32180925 PMC7053421

[ref73] Linden D.; Reddy T. B. Handbook of Batteries; The McGraw-Hill Companies, Inc, 2001; p 495.

[ref74] Shankar S.; Chorachoo J.; Jaiswal L.; Voravuthikunchai S. P. Effect of Reducing Agent Concentrations and Temperature on Characteristics and Antimicrobial Activity of Silver Nanoparticles. Mater. Lett. 2014, 137, 160–163. 10.1016/j.matlet.2014.08.100.

[ref75] Tamboli D. P.; Lee D. S. Mechanistic Antimicrobial Approach of Extracellularly Synthesized Silver Nanoparticles Against Gram Positive and Gram Negative Bacteria. J. Hazard. Mater. 2013, 260, 878–884. 10.1016/j.jhazmat.2013.06.003. 23867968

[ref76] Li Y.; Lee W.; Lee D. K.; Kim K.; Park N. G.; Ko M. J. Pure anatase TiO_2_ ″nanoglue″: An Inorganic Binding Agent to Improve Nanoparticle Interconnections in the Low-Temperature Sintering of Dye-Sensitized Solar Cells. Appl. Phys. Lett. 2011, 98, 103301 10.1063/1.3562030.

[ref77] Ryu S. Y.; Hoffmann M. R. Mixed-Metal Semiconductor Anodes for Electrochemical Water Splitting and Reactive Chlorine Species Generation: Implications for Electrochemical Wastewater Treatment. Catalysts 2016, 6, 59 10.3390/catal6040059.

