# Supplementary material for: α-NiO/Ni(OH)2/AgNP/F-Graphene Composite for Energy Storage Application
Source: ACS Omega. 2023 Mar 15;8(12):10906–18. doi: 10.1021/acsomega.2c07322 (PMC10061603; doi:10.1021/acsomega.2c07322)
Supplement: Supplementary file 1 — ao2c07322_si_001.pdf [file ao2c07322_si_001.pdf]

Supporting Information

α-NiO/Ni(OH)$_2$/AgNP/F-graphene Composite for Energy Storage Application

Su Young Ryu and Michael R. Hoffmann*

Environmental Science & Engineering
Linde Laboratory
California Institute of Technology
Pasadena, CA 91125, USA

Corresponding authors. E-mail: mrh@caltech.edu and syryu@caltech.edu

Tel: +1-626-395-4391

Fax: +1-626-395-2940

Contents:

Figure S1. The schematic illustration of the fabrication and synthesis process of α-NiO/Ni$(OH)_2$/AgNP/F-graphene composite electrode.

Figure S2. CVs of Zn (foam) - α-NiO/Ni$(OH)_2$/AgNP(200)/FG coupled electrodes obtained at the various voltage ranges after the charging/discharging process.

Figure S3. The charge/discharge voltage profiles with specific capacity that is dependent on the current density and time balance for the charging/discharging process.

Figure S4. The charge/discharge voltage profiles for the half cell electrochemical reactions of (a) α-NiO/Ni$(OH)_2$/AgNP(200)/FG - Pt in the standard 3 electrodes configuration, and (b) Zn foam (vs Pt) as reference. (c) the discharge voltage profiles of α-NiO/Ni$(OH)_2$/AgNP(200)/FG, indicating a charging time dependence. (d) the comparative CV curves of α-NiO/Ni$(OH)_2$/AgNP(200)/FG - Pt in the standard 3 electrodes configuration.

Figure S5. (A) the plot of the specific energy (W h kg^{-1}) vs time (s) for the discharging process based on the data in Figure 8A(a). (B) the representative energy draining rates for the active species of Ni and Ag especially. Each active species exhibits nearly identical energy drain rates regardless of charging current densities.

Figure S6. The portions of specific energy (W h kg^{-1}) contributed by each electrochemical reaction of Zn-Ni, Zn-Ag/O_2, and Zn-air.

Table S1. The specific energy (W h kg^{-1}) contributed by each electrochemical reaction of Zn-Ni and Zn-Ag/air and Zn-air.

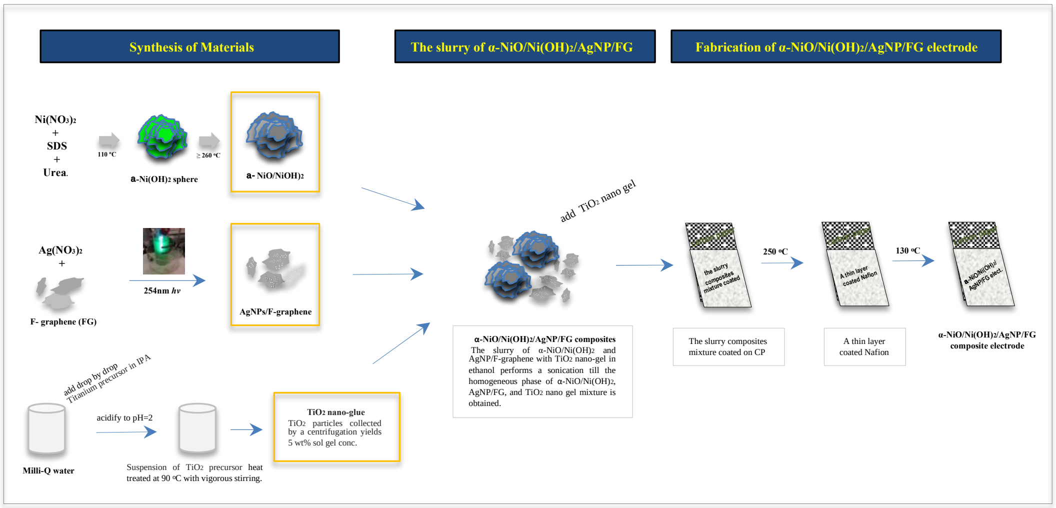

Figure S1. The schematic illustration of the fabrication and synthesis process of α-NiO/Ni(OH)$_2$/AgNP/F-graphene composite electrode.

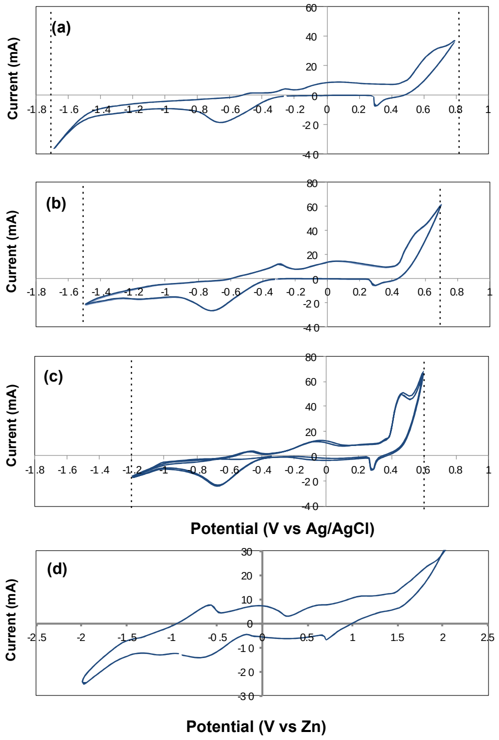

Figure S2. CVs of Zn (foam) - α-NiO/Ni(OH)$_2$/AgNP(200)/FG coupled electrodes obtained at the various voltage ranges after the charging/discharging process. The CVs of (a) ~ (c) in the standard 3 electrodes set up, and (d) in the 2 electrode configuration. The voltage scan rate was 5 mV/s.

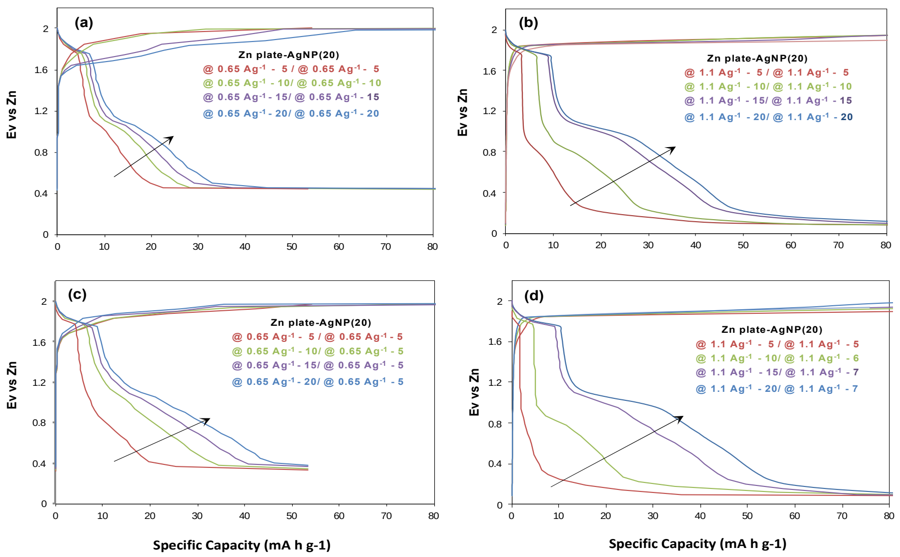

Figure S3. The charge/discharge voltage profiles with specific capacity that is dependent on the current density and time balance for the charging/discharging process. (a) and (b): the charging/discharging at the identical conditions for each cycle with increasing time in sequence of 5, 10, 15, 20 min at the current density of 0.65 and 1.1 A g^{-1}. (c) and (d): the unbalanced charging/discharging time process at the current density of 0.65 and 1.1 A g^{-1} as given a short discharging time as about 5 min, while increasing a charging time in sequence of 5, 10, 15, 20 min, respectively. The mass of active material is the total mass of AgNP + FG + α-NiO/$Ni(OH)_2$ + TiO_2.

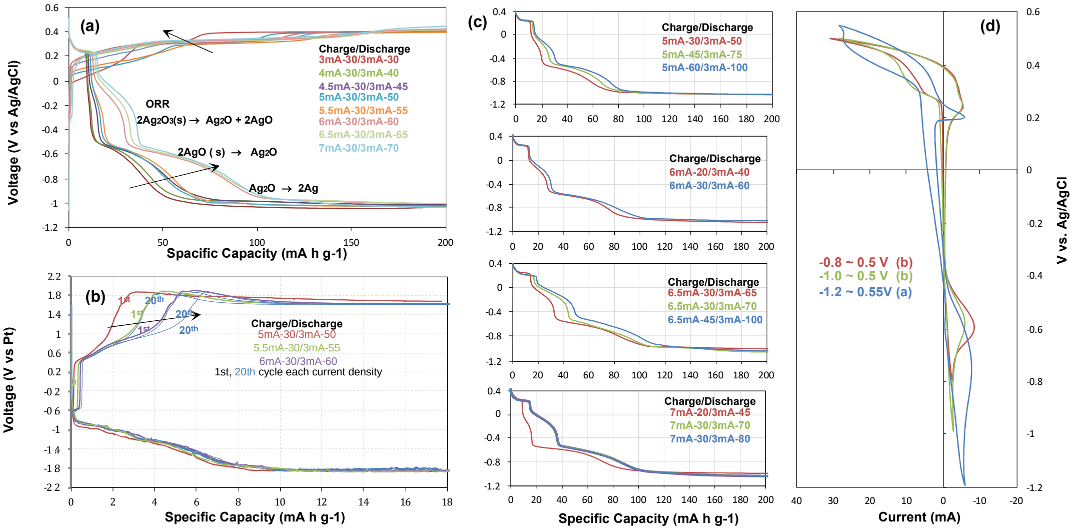

Figure S4. The charge/discharge voltage profiles for the half cell electrochemical reactions of (**a**) α-NiO/Ni(OH)$_2$/AgNP(200)/FG - Pt in the standard 3 electrodes configuration, and (**b**) Zn foam (vs Pt) as reference: (**a)** charging at the current density of 0.38 Ag^{-1}(3mA), 0.5 Ag^{-1}(4mA), 0.56 Ag^{-1} (4.5mA), 0.63 Ag^{-1} (5mA), 0.69 Ag^{-1} (5.5mA), 0.75 Ag^{-1} (6mA), 0.81 Ag^{-1}(6.5mA) and 0.88 Ag^{-1} (7mA) for 30 min, followed by discharging process at 0.38 Ag^{-1}(3mA) with time variation from 30 to 70 min in the manner of matching the charge/discharge capacity. (**b)** charging 30 min at the respective current density of 0.036 Ag^{-1}(5mA), 0.039 Ag^{-1}(5.5mA), 0.043 Ag^{-1}(6mA), followed by discharging at 0.021 Ag^{-1} (3mA) for each of 50, 55, 60 min. (**c)** the discharge voltage profiles of α-NiO/Ni(OH)$_2$/AgNP(200)/FG, indicating a charging time dependence. (**d**) the comparative CV curves of α-NiO/Ni(OH)$_2$/AgNP(200)/FG – Pt in the standard 3 electrodes configuration: (red & green) before charge/discharge cycles; (blue) after charge/discharge cycles for 123 hrs. The mass of α-NiO/Ni(OH)$_2$/AgNP(200)/FG electrode is considered as a total mass of AgNPs + FG + α-NiO/Ni(OH)$_2$ + TiO$_2$. The total mass of Zn foam is considered as a mass of active materials for the half-cell electrochemical reaction of Zn (vs Pt) in (**b**).

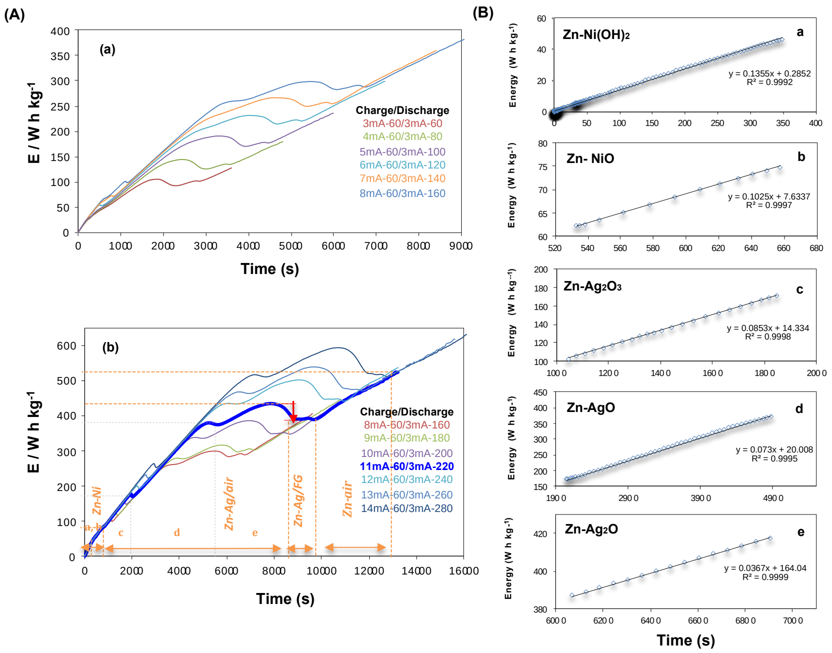

Figure S5. **(A)** the plot of the specific energy (W h kg^{-1}) vs time (s) for the discharging process based on the data in Figure 8A(a). (a**)** the discharging process after charging at the relatively low current density between 0.375 Ag^{-1} (3 mA) and 1.0 Ag^{-1}(8 mA); (b**)** the discharging process after charging at the relatively high current density between 1.0 Ag^{-1} (8 mA) and 1.75 Ag^{-1} (14 mA), respectively. **(B)** the representative energy draining rates for the active species of Ni and Ag especially, as obtained based on the blue (bold) line in (A)-b that charged at 1.375 Ag^{-1} (11 mA). Each active species exhibits nearly identical energy drain rates regardless of charging current densities.

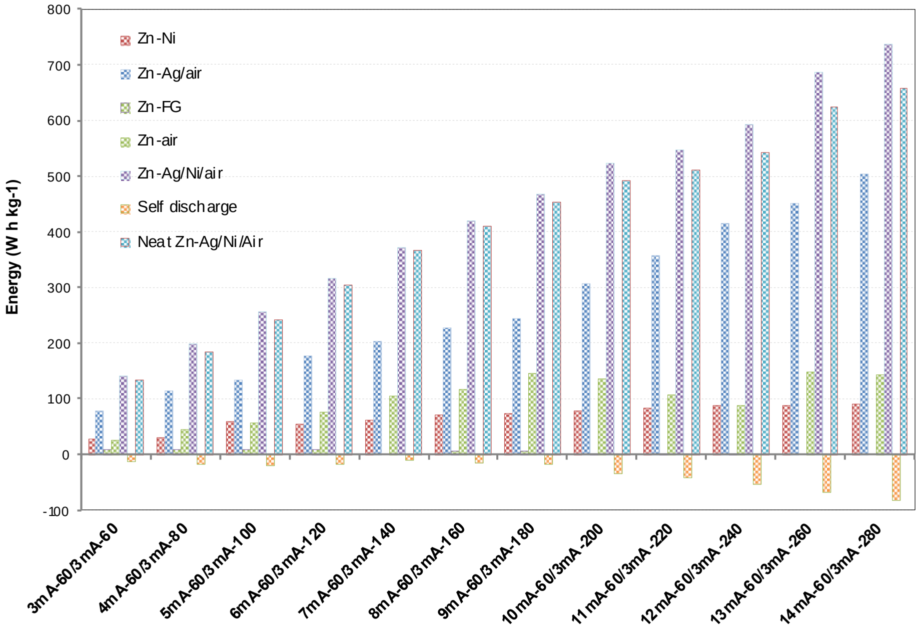

Figure S6. The portion of specific energy (W h kg^{-1}) contributed by each electrochemical reaction of Zn-Ni, Zn-Ag/O_2 and Zn-air.

Table S1. The specific energy (W h kg^{-1}) contributed by each electrochemical reaction of Zn-Ni and Zn-Ag/air and Zn-air. It contains the portion of F-graphene for ORR and the self-discharged energy detected after Zn-Ag/air electrochemical reaction. The neat specific energy is obtained by a subtraction of self-discharged energy from total energy by each electrochemical reaction.

Charge/Discharge	Zn-Ni	Zn-Ag/air	Zn-FG	Zn-air	Zn – Ni/Ag/air	Self discharge	Neat Zn-Ni//Ag/Air
3mA-60/3mA-60	27	79	9	26	141	-13	134
4mA-60/3mA-80	29	115	9	45	198	-19	185
5mA-60/3mA-100	58	133	8	57	256	-20	242
6mA-60/3mA-120	55	176	9	76	316	-17	305
7mA-60/3mA-140	62	204	-	105	371	-11	366
8mA-60/3mA-160	70	228	5	117	420	-15	410
9mA-60/3mA-180	74	243	5	145	467	-19	453
10mA-60/3mA-200	78	307	2	136	523	-36	492
11mA-60/3mA-220	84	356	1	106	547	-43	510
12mA-60/3mA-220	88	414	1	89	592	-55	542
13mA-60/3mA-260	88	451	-	147	686	-68	623
14mA-60/3mA-280	90	503	-	143	736	-82	659
